# Supplementary material for: Model guided trait-specific co-expression network estimation as a new perspective for identifying molecular interactions and pathways
Source: PLoS Comput Biol. 2021 May 3;17(5):e1008960. doi: 10.1371/journal.pcbi.1008960 (PMC8118548; doi:10.1371/journal.pcbi.1008960)
Supplement: S2 Appendix — See also a GitHub repository https://github.com/JAJKontio/model_diffnet.git. (ZIP) [file pcbi.1008960.s002.zip › Notes_leukemia_analysis.nb.html]

Acute myeloid leukemia analysis: R-code


Code 

- Show All Code
- Hide All Code
- Download Rmd

# Acute myeloid leukemia analysis: R-code

Go to a website (https://www.synapse.org/#!Synapse:syn2455683/wiki/64007) and download a normalized protein expression DREAM9-challenge dataset (trainingData-release.csv) (Noren et al.2016 - https://doi.org/10.1371/journal.pcbi.1004890) which is available upon registration (http://dreamchallenges.org/). These data were provided by Dr. Steven Kornblau from the University of Texas MD Anderson Cancer Center and were obtained through Synapse syn2455683 as a part of the acute myeloid leukemia (AML) DREAM-challenge.

#STEP 0: PREPARING THE DATASET AND INSTALLING THE REQUIRED R-PACKAGES Install the required packages and prepare the AML dataset from the downloaded CSV-file “trainingData-release.csv” (requires registration) - https://www.synapse.org/#!Synapse:syn2455683/wiki/64007.


```
#DOWNLOAD REQUIRED R-PACKAGES
library("glmnet") #https://CRAN.R-project.org/package=glmnet
library("matrixStats") #https://CRAN.R-project.org/package=matrixStats
library("qgraph") #https://CRAN.R-project.org/package=qgraph 
library("netdiffuseR") #https://CRAN.R-project.org/package=netdiffuseR
library("stringr") #https://CRAN.R-project.org/package=stringr 

#Replace "~/trainingData-release.csv" with a user-specific path to the downloaded CSV-file.
data <-read.csv("~/trainingData-release.csv", header = TRUE)
attach(data)

#Transform measured survival times ("Overall_Survival" variable) into a logarithmic scale.
logT <- log(Overall_Survival)

#Separate the expression data (renamed as "rdata") from clinical covariates and convert the censoring variable, "vital.status", to a numeric binary vector (deceased -> 1 and alive -> 0).
rdata <- data[,c(42:272)]
censor <- as.vector(vital.status)
censor[censor == "D"] <- 1
censor[censor == "A"] <- 0
censor <- as.numeric(censor)

#Segregate non-censored observations for the following two steps. These omitted samples are taken account for in the forthcoming re-evaluations steps / see the paper for detailed discussion.
rdata <- rdata[which(censor == 1),]
rdata <- as.matrix(rdata)
logT <- logT[which(censor==1)]
```


#STEP 1: Estimate the residual vector from the main effect model (See Equation 3). In this step, the main effects are estimated with the elastic-net algorithm using the “glmnet” R-package (https://CRAN.R-project.org/package=glmnet), where an optimal penalty parameter value is chosen by 5-fold cross-validation.


```
#An argument "alpha" is the elastic net mixing parameter which gives the LASSO penalty with alpha = 1, and the ridge penalty with alpha = 0. Thus, the ridge-penalty is slightly more emphasized, meaning that the algorithm is, in comparison to the LASSO-penalty, more inclined to pick or eliminate highly correlated explanatory variables simultaneously instead of randomly picking and focusing only one of them (a known property of the LASSO algorithm).
cv.fit <- cv.glmnet(rdata, scale(logT), alpha = 1/3, maxit = 10000)
fit=glmnet(rdata, scale(logT), alpha = 1/3, maxit = 10000)

#The resulted lambda value was 0.4536807 in our cross-validation run. 
Coefficients <- coef(fit, s = 0.4536807)
#*Use this line for new analyses* -> # Coefficients <- coef(fit, s = cv.fit$lambda.min)

#Compute the residual vector "resid" as in Equation 3.
v <- rdata %*% Coefficients[-1]
resid <- scale(logT) - v
```


#STEP 2: Estimate the high and low networks: Differential network estimation step (See also Figure 2). Here we estimate the differential network between 1) the high network, which is constructed over individuals to whom the estimated residual values are above the median value of the “resid”-variable, and 2) the low network, which is estimated over individuals with “resid”-variable values smaller the median, respectively. To estimate the corresponding network edge weights (PCCN or CCN matrix elements) by accounting for the objectives discussed in the paper, we only apply the proposed part-correlation metric (PCCN) in this analysis.


```
#Define how the high and low groups of individuals are divided from the residual vector using the cut-off point a. In our analysis, a = 1/2, which corresponds to the median of all estimated residual values, and thereby, divides the dataset in half. Note that all samples are used only with a = 1/2.
a = 1/2
#An element of the high or low network is deemed to be zero in the estimation process if its magnitude is below the value of r >= 0. Note that a non-zero value is required for identifying type II interactions.
r = 0.1

#The following loop estimates the differential part-correlation matrix (dPCCN) elements and assign their signs into an another matrix in order to compute the sign-adjusted version (signdPCCN).
signP <- dPCCN  <- matrix(0,ncol(rdata),ncol(rdata))

for(i in 1:ncol(rdata)){
  for(j in 1:ncol(rdata)){
    
    res <- (lm(rdata[,j]~rdata[,i])$residuals)
    set <- cbind(resid,res,rdata[,i])
    high <- set[which(resid > quantile(resid, a)),]
    low <- set[which(resid < quantile(resid,1-a)),]
    high <- high[,-1]; low <- low[,-1]
    
    if(abs(cor(high[,1],high[,2])) < r){
      H <- 0
    }else{
      H <- cor(high[,1],high[,2])
    }
    
    if(abs(cor(low[,1],low[,2])) < r){
      L <- 0
    }else{
      L <- cor(low[,1],low[,2])
    }
    
    dPCCN[i,j] <- H-L
    
    signP[i,j] <- 0.5*abs((sign(H)-sign(L)))
    
     }
}
```


#STEP 3: Plot and link the network connections with the underlying parametric model. The following lines plot two separate networks corresponding to type I and II interactions.


```
#Assign negative values for type II interactions (+- to separate them by different colors from type I interactions in qgraph plots).
signP[signP == 0.5] <- -1
signdPCCN <- signP*abs(dPCCN)

#The desired number of non-zero network elements (see the paper) are given by a hard-thresholding procedure.
diag(signdPCCN) <- 0
graph <- signdPCCN
graph[which(graph < 0.56  & graph > -0.155)] <- 0
for(i in 1:ncol(rdata)){
  for(j in 1:ncol(rdata)){
    if(graph[i,j] == 0 | graph[j,i] == 0){
      graph[i,j] <- 0
      graph[j,i] <- 0
    }
  }
}

#PLOT FIGURE 3.
graphplus <- graphminus <- graph

#Separate positive and negative network elements.
graphplus[which(graphplus < 0)] <- 0
graphminus[which(graphminus > 0)] <- 0
graphplus[lower.tri(graphplus)] <- 0
graphminus[lower.tri(graphminus)] <- 0

#Create symmetric matrices.
graphplus <- graphplus + t(graphplus)
graphminus <-graphminus + t(graphminus)
 
#Remove all unconnected nodes from the network.
colnames(rdata) <- str_sub(colnames(rdata), start=1, end=8)
iso_plus <- isolated(as.dgCMatrix(graphplus),undirected = TRUE)
connected_plus <- colnames(rdata)[which(iso_plus == "FALSE")]
iso_minus <- isolated(as.dgCMatrix(graphminus),undirected =TRUE)
connected_minus <- colnames(rdata)[which(iso_minus == "FALSE")]

#Remove the network "weights" by assigning the same values for all non-zero components within both networks.
graphplus[which(graphplus != 0)] <- 1
graphminus[which(graphminus != 0)] <- -1

#Plot the resulting networks using the "qgraph" function. 

par(mfrow=c(1,2))
qgraph(graphplus[which(iso_plus == "FALSE"),which(iso_plus == "FALSE")], labels=colnames(rdata)[which(iso_plus == "FALSE")],vsize = c(5, 5), esize = 4,curve = 3, layout="spring",title = "Type I interactions",edge.color="darkgreen", label.fill.horizontal =1,label.fill.vertical=1,label.prop =0.93,title.cex = 1.45)
qgraph(graphminus[which(iso_minus == "FALSE"),which(iso_minus == "FALSE")], labels=colnames(rdata)[which(iso_minus == "FALSE")],vsize = c(5, 5), esize = 4,curve = 3,layout="spring", title = "Type II interactions",edge.color="red",label.prop =0.93,title.cex = 1.45)
```


LS0tDQp0aXRsZTogIkFjdXRlIG15ZWxvaWQgbGV1a2VtaWEgYW5hbHlzaXM6IFItY29kZSINCm91dHB1dDogaHRtbF9ub3RlYm9vaw0KLS0tIA0KR28gdG8gYSB3ZWJzaXRlIChodHRwczovL3d3dy5zeW5hcHNlLm9yZy8jIVN5bmFwc2U6c3luMjQ1NTY4My93aWtpLzY0MDA3KSBhbmQgZG93bmxvYWQgYSBub3JtYWxpemVkIHByb3RlaW4gZXhwcmVzc2lvbiBEUkVBTTktY2hhbGxlbmdlIGRhdGFzZXQgKHRyYWluaW5nRGF0YS1yZWxlYXNlLmNzdikgKE5vcmVuIGV0IGFsLjIwMTYgLSBodHRwczovL2RvaS5vcmcvMTAuMTM3MS9qb3VybmFsLnBjYmkuMTAwNDg5MCkgd2hpY2ggaXMgYXZhaWxhYmxlIHVwb24gcmVnaXN0cmF0aW9uIChodHRwOi8vZHJlYW1jaGFsbGVuZ2VzLm9yZy8pLiBUaGVzZSBkYXRhIHdlcmUgcHJvdmlkZWQgYnkgRHIuIFN0ZXZlbiBLb3JuYmxhdSBmcm9tIHRoZSBVbml2ZXJzaXR5IG9mIFRleGFzIE1EIEFuZGVyc29uIENhbmNlciBDZW50ZXIgYW5kIHdlcmUgb2J0YWluZWQgdGhyb3VnaCBTeW5hcHNlIHN5bjI0NTU2ODMgYXMgYSBwYXJ0IG9mIHRoZSBhY3V0ZSBteWVsb2lkIGxldWtlbWlhIChBTUwpIERSRUFNLWNoYWxsZW5nZS4gDQoNCiNTVEVQIDA6IFBSRVBBUklORyBUSEUgREFUQVNFVCBBTkQgSU5TVEFMTElORyBUSEUgUkVRVUlSRUQgUi1QQUNLQUdFUw0KSW5zdGFsbCB0aGUgcmVxdWlyZWQgcGFja2FnZXMgYW5kIHByZXBhcmUgdGhlIEFNTCBkYXRhc2V0IGZyb20gdGhlIGRvd25sb2FkZWQgQ1NWLWZpbGUgInRyYWluaW5nRGF0YS1yZWxlYXNlLmNzdiIgKHJlcXVpcmVzIHJlZ2lzdHJhdGlvbikgLSBodHRwczovL3d3dy5zeW5hcHNlLm9yZy8jIVN5bmFwc2U6c3luMjQ1NTY4My93aWtpLzY0MDA3Lg0KDQpgYGB7cn0NCiNET1dOTE9BRCBSRVFVSVJFRCBSLVBBQ0tBR0VTDQpsaWJyYXJ5KCJnbG1uZXQiKSAjaHR0cHM6Ly9DUkFOLlItcHJvamVjdC5vcmcvcGFja2FnZT1nbG1uZXQNCmxpYnJhcnkoIm1hdHJpeFN0YXRzIikgI2h0dHBzOi8vQ1JBTi5SLXByb2plY3Qub3JnL3BhY2thZ2U9bWF0cml4U3RhdHMNCmxpYnJhcnkoInFncmFwaCIpICNodHRwczovL0NSQU4uUi1wcm9qZWN0Lm9yZy9wYWNrYWdlPXFncmFwaCANCmxpYnJhcnkoIm5ldGRpZmZ1c2VSIikgI2h0dHBzOi8vQ1JBTi5SLXByb2plY3Qub3JnL3BhY2thZ2U9bmV0ZGlmZnVzZVINCmxpYnJhcnkoInN0cmluZ3IiKSAjaHR0cHM6Ly9DUkFOLlItcHJvamVjdC5vcmcvcGFja2FnZT1zdHJpbmdyIA0KDQojUmVwbGFjZSAifi90cmFpbmluZ0RhdGEtcmVsZWFzZS5jc3YiIHdpdGggYSB1c2VyLXNwZWNpZmljIHBhdGggdG8gdGhlIGRvd25sb2FkZWQgQ1NWLWZpbGUuDQpkYXRhIDwtcmVhZC5jc3YoIn4vdHJhaW5pbmdEYXRhLXJlbGVhc2UuY3N2IiwgaGVhZGVyID0gVFJVRSkNCmF0dGFjaChkYXRhKQ0KDQojVHJhbnNmb3JtIG1lYXN1cmVkIHN1cnZpdmFsIHRpbWVzICgiT3ZlcmFsbF9TdXJ2aXZhbCIgdmFyaWFibGUpIGludG8gYSBsb2dhcml0aG1pYyBzY2FsZS4NCmxvZ1QgPC0gbG9nKE92ZXJhbGxfU3Vydml2YWwpDQoNCiNTZXBhcmF0ZSB0aGUgZXhwcmVzc2lvbiBkYXRhIChyZW5hbWVkIGFzICJyZGF0YSIpIGZyb20gY2xpbmljYWwgY292YXJpYXRlcyBhbmQgY29udmVydCB0aGUgY2Vuc29yaW5nIHZhcmlhYmxlLCAidml0YWwuc3RhdHVzIiwgdG8gYSBudW1lcmljIGJpbmFyeSB2ZWN0b3IgKGRlY2Vhc2VkIC0+IDEgYW5kIGFsaXZlIC0+IDApLg0KcmRhdGEgPC0gZGF0YVssYyg0MjoyNzIpXQ0KY2Vuc29yIDwtIGFzLnZlY3Rvcih2aXRhbC5zdGF0dXMpDQpjZW5zb3JbY2Vuc29yID09ICJEIl0gPC0gMQ0KY2Vuc29yW2NlbnNvciA9PSAiQSJdIDwtIDANCmNlbnNvciA8LSBhcy5udW1lcmljKGNlbnNvcikNCg0KI1NlZ3JlZ2F0ZSBub24tY2Vuc29yZWQgb2JzZXJ2YXRpb25zIGZvciB0aGUgZm9sbG93aW5nIHR3byBzdGVwcy4gVGhlc2Ugb21pdHRlZCBzYW1wbGVzIGFyZSB0YWtlbiBhY2NvdW50IGZvciBpbiB0aGUgZm9ydGhjb21pbmcgcmUtZXZhbHVhdGlvbnMgc3RlcHMgLyBzZWUgdGhlIHBhcGVyIGZvciBkZXRhaWxlZCBkaXNjdXNzaW9uLg0KcmRhdGEgPC0gcmRhdGFbd2hpY2goY2Vuc29yID09IDEpLF0NCnJkYXRhIDwtIGFzLm1hdHJpeChyZGF0YSkNCmxvZ1QgPC0gbG9nVFt3aGljaChjZW5zb3I9PTEpXQ0KYGBgDQojU1RFUCAxOiBFc3RpbWF0ZSB0aGUgcmVzaWR1YWwgdmVjdG9yIGZyb20gdGhlIG1haW4gZWZmZWN0IG1vZGVsIChTZWUgRXF1YXRpb24gMykuIA0KSW4gdGhpcyBzdGVwLCB0aGUgbWFpbiBlZmZlY3RzIGFyZSBlc3RpbWF0ZWQgd2l0aCB0aGUgZWxhc3RpYy1uZXQgYWxnb3JpdGhtIHVzaW5nIHRoZSAiZ2xtbmV0IiBSLXBhY2thZ2UgKGh0dHBzOi8vQ1JBTi5SLXByb2plY3Qub3JnL3BhY2thZ2U9Z2xtbmV0KSwgd2hlcmUgYW4gb3B0aW1hbCBwZW5hbHR5IHBhcmFtZXRlciB2YWx1ZSBpcyBjaG9zZW4gYnkgNS1mb2xkIGNyb3NzLXZhbGlkYXRpb24uDQpgYGB7cn0NCiNBbiBhcmd1bWVudCAiYWxwaGEiIGlzIHRoZSBlbGFzdGljIG5ldCBtaXhpbmcgcGFyYW1ldGVyIHdoaWNoIGdpdmVzIHRoZSBMQVNTTyBwZW5hbHR5IHdpdGggYWxwaGEgPSAxLCBhbmQgdGhlIHJpZGdlIHBlbmFsdHkgd2l0aCBhbHBoYSA9IDAuIFRodXMsIHRoZSByaWRnZS1wZW5hbHR5IGlzIHNsaWdodGx5IG1vcmUgZW1waGFzaXplZCwgbWVhbmluZyB0aGF0IHRoZSBhbGdvcml0aG0gaXMsIGluIGNvbXBhcmlzb24gdG8gdGhlIExBU1NPLXBlbmFsdHksIG1vcmUgaW5jbGluZWQgdG8gcGljayBvciBlbGltaW5hdGUgaGlnaGx5IGNvcnJlbGF0ZWQgZXhwbGFuYXRvcnkgdmFyaWFibGVzIHNpbXVsdGFuZW91c2x5IGluc3RlYWQgb2YgcmFuZG9tbHkgcGlja2luZyBhbmQgZm9jdXNpbmcgb25seSBvbmUgb2YgdGhlbSAoYSBrbm93biBwcm9wZXJ0eSBvZiB0aGUgTEFTU08gYWxnb3JpdGhtKS4NCmN2LmZpdCA8LSBjdi5nbG1uZXQocmRhdGEsIHNjYWxlKGxvZ1QpLCBhbHBoYSA9IDEvMywgbWF4aXQgPSAxMDAwMCkNCmZpdD1nbG1uZXQocmRhdGEsIHNjYWxlKGxvZ1QpLCBhbHBoYSA9IDEvMywgbWF4aXQgPSAxMDAwMCkNCg0KI1RoZSByZXN1bHRlZCBsYW1iZGEgdmFsdWUgd2FzIDAuNDUzNjgwNyBpbiBvdXIgY3Jvc3MtdmFsaWRhdGlvbiBydW4uIA0KQ29lZmZpY2llbnRzIDwtIGNvZWYoZml0LCBzID0gMC40NTM2ODA3KQ0KIypVc2UgdGhpcyBsaW5lIGZvciBuZXcgYW5hbHlzZXMqIC0+ICMgQ29lZmZpY2llbnRzIDwtIGNvZWYoZml0LCBzID0gY3YuZml0JGxhbWJkYS5taW4pDQoNCiNDb21wdXRlIHRoZSByZXNpZHVhbCB2ZWN0b3IgInJlc2lkIiBhcyBpbiBFcXVhdGlvbiAzLg0KdiA8LSByZGF0YSAlKiUgQ29lZmZpY2llbnRzWy0xXQ0KcmVzaWQgPC0gc2NhbGUobG9nVCkgLSB2DQpgYGANCiNTVEVQIDI6IEVzdGltYXRlIHRoZSBoaWdoIGFuZCBsb3cgbmV0d29ya3M6IERpZmZlcmVudGlhbCBuZXR3b3JrIGVzdGltYXRpb24gc3RlcCAoU2VlIGFsc28gRmlndXJlIDIpLg0KSGVyZSB3ZSBlc3RpbWF0ZSB0aGUgZGlmZmVyZW50aWFsIG5ldHdvcmsgYmV0d2VlbiAxKSB0aGUgaGlnaCBuZXR3b3JrLCB3aGljaCBpcyBjb25zdHJ1Y3RlZCBvdmVyIGluZGl2aWR1YWxzIHRvIHdob20gdGhlIGVzdGltYXRlZCByZXNpZHVhbCB2YWx1ZXMgYXJlIGFib3ZlIHRoZSBtZWRpYW4gdmFsdWUgb2YgdGhlICJyZXNpZCItdmFyaWFibGUsIGFuZCAyKSB0aGUgbG93IG5ldHdvcmssIHdoaWNoIGlzIGVzdGltYXRlZCBvdmVyIGluZGl2aWR1YWxzIHdpdGggInJlc2lkIi12YXJpYWJsZSB2YWx1ZXMgc21hbGxlciB0aGUgIG1lZGlhbiwgcmVzcGVjdGl2ZWx5LiBUbyBlc3RpbWF0ZSB0aGUgY29ycmVzcG9uZGluZyBuZXR3b3JrIGVkZ2Ugd2VpZ2h0cyAoUENDTiBvciBDQ04gbWF0cml4IGVsZW1lbnRzKSBieSBhY2NvdW50aW5nIGZvciB0aGUgb2JqZWN0aXZlcyBkaXNjdXNzZWQgaW4gdGhlIHBhcGVyLCB3ZSBvbmx5IGFwcGx5IHRoZSBwcm9wb3NlZCBwYXJ0LWNvcnJlbGF0aW9uIG1ldHJpYyAoUENDTikgaW4gdGhpcyBhbmFseXNpcy4gDQpgYGB7cn0NCiNEZWZpbmUgaG93IHRoZSBoaWdoIGFuZCBsb3cgZ3JvdXBzIG9mIGluZGl2aWR1YWxzIGFyZSBkaXZpZGVkIGZyb20gdGhlIHJlc2lkdWFsIHZlY3RvciB1c2luZyB0aGUgY3V0LW9mZiBwb2ludCBhLiBJbiBvdXIgYW5hbHlzaXMsIGEgPSAxLzIsIHdoaWNoIGNvcnJlc3BvbmRzIHRvIHRoZSBtZWRpYW4gb2YgYWxsIGVzdGltYXRlZCByZXNpZHVhbCB2YWx1ZXMsIGFuZCB0aGVyZWJ5LCBkaXZpZGVzIHRoZSBkYXRhc2V0IGluIGhhbGYuIE5vdGUgdGhhdCBhbGwgc2FtcGxlcyBhcmUgdXNlZCBvbmx5IHdpdGggYSA9IDEvMi4NCmEgPSAxLzINCiNBbiBlbGVtZW50IG9mIHRoZSBoaWdoIG9yIGxvdyBuZXR3b3JrIGlzIGRlZW1lZCB0byBiZSB6ZXJvIGluIHRoZSBlc3RpbWF0aW9uIHByb2Nlc3MgaWYgaXRzIG1hZ25pdHVkZSBpcyBiZWxvdyB0aGUgdmFsdWUgb2YgciA+PSAwLiBOb3RlIHRoYXQgYSBub24temVybyB2YWx1ZSBpcyByZXF1aXJlZCBmb3IgaWRlbnRpZnlpbmcgdHlwZSBJSSBpbnRlcmFjdGlvbnMuDQpyID0gMC4xDQoNCiNUaGUgZm9sbG93aW5nIGxvb3AgZXN0aW1hdGVzIHRoZSBkaWZmZXJlbnRpYWwgcGFydC1jb3JyZWxhdGlvbiBtYXRyaXggKGRQQ0NOKSBlbGVtZW50cyBhbmQgYXNzaWduIHRoZWlyIHNpZ25zIGludG8gYW4gYW5vdGhlciBtYXRyaXggaW4gb3JkZXIgdG8gY29tcHV0ZSB0aGUgc2lnbi1hZGp1c3RlZCB2ZXJzaW9uIChzaWduZFBDQ04pLg0Kc2lnblAgPC0gZFBDQ04gIDwtIG1hdHJpeCgwLG5jb2wocmRhdGEpLG5jb2wocmRhdGEpKQ0KDQpmb3IoaSBpbiAxOm5jb2wocmRhdGEpKXsNCiAgZm9yKGogaW4gMTpuY29sKHJkYXRhKSl7DQogICAgDQogICAgcmVzIDwtIChsbShyZGF0YVssal1+cmRhdGFbLGldKSRyZXNpZHVhbHMpDQogICAgc2V0IDwtIGNiaW5kKHJlc2lkLHJlcyxyZGF0YVssaV0pDQogICAgaGlnaCA8LSBzZXRbd2hpY2gocmVzaWQgPiBxdWFudGlsZShyZXNpZCwgYSkpLF0NCiAgICBsb3cgPC0gc2V0W3doaWNoKHJlc2lkIDwgcXVhbnRpbGUocmVzaWQsMS1hKSksXQ0KICAgIGhpZ2ggPC0gaGlnaFssLTFdOyBsb3cgPC0gbG93WywtMV0NCiAgICANCiAgICBpZihhYnMoY29yKGhpZ2hbLDFdLGhpZ2hbLDJdKSkgPCByKXsNCiAgICAgIEggPC0gMA0KICAgIH1lbHNlew0KICAgICAgSCA8LSBjb3IoaGlnaFssMV0saGlnaFssMl0pDQogICAgfQ0KICAgIA0KICAgIGlmKGFicyhjb3IobG93WywxXSxsb3dbLDJdKSkgPCByKXsNCiAgICAgIEwgPC0gMA0KICAgIH1lbHNlew0KICAgICAgTCA8LSBjb3IobG93WywxXSxsb3dbLDJdKQ0KICAgIH0NCiAgICANCiAgICBkUENDTltpLGpdIDwtIEgtTA0KICAgIA0KICAgIHNpZ25QW2ksal0gPC0gMC41KmFicygoc2lnbihIKS1zaWduKEwpKSkNCiAgICANCiAgICAgfQ0KfQ0KYGBgDQojU1RFUCAzOiBQbG90IGFuZCBsaW5rIHRoZSBuZXR3b3JrIGNvbm5lY3Rpb25zIHdpdGggdGhlIHVuZGVybHlpbmcgcGFyYW1ldHJpYyBtb2RlbC4NClRoZSBmb2xsb3dpbmcgbGluZXMgcGxvdCB0d28gc2VwYXJhdGUgbmV0d29ya3MgY29ycmVzcG9uZGluZyB0byB0eXBlIEkgYW5kIElJIGludGVyYWN0aW9ucy4NCmBgYHtyfQ0KI0Fzc2lnbiBuZWdhdGl2ZSB2YWx1ZXMgZm9yIHR5cGUgSUkgaW50ZXJhY3Rpb25zICgrLSB0byBzZXBhcmF0ZSB0aGVtIGJ5IGRpZmZlcmVudCBjb2xvcnMgZnJvbSB0eXBlIEkgaW50ZXJhY3Rpb25zIGluIHFncmFwaCBwbG90cykuDQpzaWduUFtzaWduUCA9PSAwLjVdIDwtIC0xDQpzaWduZFBDQ04gPC0gc2lnblAqYWJzKGRQQ0NOKQ0KDQojVGhlIGRlc2lyZWQgbnVtYmVyIG9mIG5vbi16ZXJvIG5ldHdvcmsgZWxlbWVudHMgKHNlZSB0aGUgcGFwZXIpIGFyZSBnaXZlbiBieSBhIGhhcmQtdGhyZXNob2xkaW5nIHByb2NlZHVyZS4NCmRpYWcoc2lnbmRQQ0NOKSA8LSAwDQpncmFwaCA8LSBzaWduZFBDQ04NCmdyYXBoW3doaWNoKGdyYXBoIDwgMC41NiAgJiBncmFwaCA+IC0wLjE1NSldIDwtIDANCmZvcihpIGluIDE6bmNvbChyZGF0YSkpew0KICBmb3IoaiBpbiAxOm5jb2wocmRhdGEpKXsNCiAgICBpZihncmFwaFtpLGpdID09IDAgfCBncmFwaFtqLGldID09IDApew0KICAgICAgZ3JhcGhbaSxqXSA8LSAwDQogICAgICBncmFwaFtqLGldIDwtIDANCiAgICB9DQogIH0NCn0NCg0KI1BMT1QgRklHVVJFIDMuDQpncmFwaHBsdXMgPC0gZ3JhcGhtaW51cyA8LSBncmFwaA0KDQojU2VwYXJhdGUgcG9zaXRpdmUgYW5kIG5lZ2F0aXZlIG5ldHdvcmsgZWxlbWVudHMuDQpncmFwaHBsdXNbd2hpY2goZ3JhcGhwbHVzIDwgMCldIDwtIDANCmdyYXBobWludXNbd2hpY2goZ3JhcGhtaW51cyA+IDApXSA8LSAwDQpncmFwaHBsdXNbbG93ZXIudHJpKGdyYXBocGx1cyldIDwtIDANCmdyYXBobWludXNbbG93ZXIudHJpKGdyYXBobWludXMpXSA8LSAwDQoNCiNDcmVhdGUgc3ltbWV0cmljIG1hdHJpY2VzLg0KZ3JhcGhwbHVzIDwtIGdyYXBocGx1cyArIHQoZ3JhcGhwbHVzKQ0KZ3JhcGhtaW51cyA8LWdyYXBobWludXMgKyB0KGdyYXBobWludXMpDQogDQojUmVtb3ZlIGFsbCB1bmNvbm5lY3RlZCBub2RlcyBmcm9tIHRoZSBuZXR3b3JrLg0KY29sbmFtZXMocmRhdGEpIDwtIHN0cl9zdWIoY29sbmFtZXMocmRhdGEpLCBzdGFydD0xLCBlbmQ9OCkNCmlzb19wbHVzIDwtIGlzb2xhdGVkKGFzLmRnQ01hdHJpeChncmFwaHBsdXMpLHVuZGlyZWN0ZWQgPSBUUlVFKQ0KY29ubmVjdGVkX3BsdXMgPC0gY29sbmFtZXMocmRhdGEpW3doaWNoKGlzb19wbHVzID09ICJGQUxTRSIpXQ0KaXNvX21pbnVzIDwtIGlzb2xhdGVkKGFzLmRnQ01hdHJpeChncmFwaG1pbnVzKSx1bmRpcmVjdGVkID1UUlVFKQ0KY29ubmVjdGVkX21pbnVzIDwtIGNvbG5hbWVzKHJkYXRhKVt3aGljaChpc29fbWludXMgPT0gIkZBTFNFIildDQoNCiNSZW1vdmUgdGhlIG5ldHdvcmsgIndlaWdodHMiIGJ5IGFzc2lnbmluZyB0aGUgc2FtZSB2YWx1ZXMgZm9yIGFsbCBub24temVybyBjb21wb25lbnRzIHdpdGhpbiBib3RoIG5ldHdvcmtzLg0KZ3JhcGhwbHVzW3doaWNoKGdyYXBocGx1cyAhPSAwKV0gPC0gMQ0KZ3JhcGhtaW51c1t3aGljaChncmFwaG1pbnVzICE9IDApXSA8LSAtMQ0KDQojUGxvdCB0aGUgcmVzdWx0aW5nIG5ldHdvcmtzIHVzaW5nIHRoZSAicWdyYXBoIiBmdW5jdGlvbi4gDQoNCnBhcihtZnJvdz1jKDEsMikpDQpxZ3JhcGgoZ3JhcGhwbHVzW3doaWNoKGlzb19wbHVzID09ICJGQUxTRSIpLHdoaWNoKGlzb19wbHVzID09ICJGQUxTRSIpXSwgbGFiZWxzPWNvbG5hbWVzKHJkYXRhKVt3aGljaChpc29fcGx1cyA9PSAiRkFMU0UiKV0sdnNpemUgPSBjKDUsIDUpLCBlc2l6ZSA9IDQsY3VydmUgPSAzLCBsYXlvdXQ9InNwcmluZyIsdGl0bGUgPSAiVHlwZSBJIGludGVyYWN0aW9ucyIsZWRnZS5jb2xvcj0iZGFya2dyZWVuIiwgbGFiZWwuZmlsbC5ob3Jpem9udGFsID0xLGxhYmVsLmZpbGwudmVydGljYWw9MSxsYWJlbC5wcm9wID0wLjkzLHRpdGxlLmNleCA9IDEuNDUpDQpxZ3JhcGgoZ3JhcGhtaW51c1t3aGljaChpc29fbWludXMgPT0gIkZBTFNFIiksd2hpY2goaXNvX21pbnVzID09ICJGQUxTRSIpXSwgbGFiZWxzPWNvbG5hbWVzKHJkYXRhKVt3aGljaChpc29fbWludXMgPT0gIkZBTFNFIildLHZzaXplID0gYyg1LCA1KSwgZXNpemUgPSA0LGN1cnZlID0gMyxsYXlvdXQ9InNwcmluZyIsIHRpdGxlID0gIlR5cGUgSUkgaW50ZXJhY3Rpb25zIixlZGdlLmNvbG9yPSJyZWQiLGxhYmVsLnByb3AgPTAuOTMsdGl0bGUuY2V4ID0gMS40NSkNCmBgYA0KDQo=
