# Supplementary material for: Model guided trait-specific co-expression network estimation as a new perspective for identifying molecular interactions and pathways
Source: PLoS Comput Biol. 2021 May 3;17(5):e1008960. doi: 10.1371/journal.pcbi.1008960 (PMC8118548; doi:10.1371/journal.pcbi.1008960)
Supplement: S2 Appendix — See also a GitHub repository https://github.com/JAJKontio/model_diffnet.git. (ZIP) [file pcbi.1008960.s002.zip › Preview_documents/SIMULATED_EXAMPLES_MODEL_A.nb.html]

SIMULATED EXAMPLES: MODEL A


Code 

- Show All Code
- Hide All Code
- Download Rmd

# SIMULATED EXAMPLES: MODEL A

Go to a website (https://www.synapse.org/#!Synapse:syn2455683/wiki/64007) and download a normalized protein expression DREAM9-challenge dataset (trainingData-release.csv) (Noren et al.2016 - https://doi.org/10.1371/journal.pcbi.1004890) which is available upon registration (http://dreamchallenges.org/). These data were provided by Dr. Steven Kornblau from the University of Texas MD Anderson Cancer Center and were obtained through Synapse syn2455683 as a part of the acute myeloid leukemia (AML) DREAM-challenge.

#STEP 0: PREPARING THE DATASET AND INSTALLING THE REQUIRED R-PACKAGES Install the required packages and prepare the AML dataset from the downloaded CSV-file “trainingData-release.csv” (requires registration) - https://www.synapse.org/#!Synapse:syn2455683/wiki/64007.


```
#DOWNLOAD REQUIRED R-PACKAGES
library("glmnet") #https://CRAN.R-project.org/package=glmnet
library("matrixStats") #https://CRAN.R-project.org/package=matrixStats
library("qgraph") #https://CRAN.R-project.org/package=qgraph 
library("netdiffuseR") #https://CRAN.R-project.org/package=netdiffuseR
library("stringr") #https://CRAN.R-project.org/package=stringr 

#Replace "~/trainingData-release.csv" with a user-specific path to the downloaded CSV-file.
data <-read.csv("~/trainingData-release.csv", header = TRUE)

#Separate the expression data (renamed as "rdata") from clinical covariates.
rdata <- data[,c(42:272)]
```


#INITIALIZE


```
#Number of replicates
k = 10
data <- rdata
#Empty list and vectors for replicates
dCCNlist <- rep(list(matrix(NA, ncol(rdata), ncol(rdata)), k))
dPCCNlist <- rep(list(matrix(NA, ncol(rdata), ncol(rdata)), k))
GGMlist <- rep(list(matrix(NA, ncol(rdata), ncol(rdata)), k))
ymat <- matrix(0,191,k)
her1 <- rep(0,k)
rdatareps <- rep(list(matrix(NA, ncol(rdata), ncol(rdata))), k)

#Empty vectors for AUCS and their confidence intervals calculated over replicates

dCCNauc <- rep(0,k)
dPCCNauc<- rep(0,k)
signdCCNauc<- rep(0,k)
signdPCCNauc<- rep(0,k)
exhaustiveauc <- rep(0,k)
dGGMauc <- rep(0,k)

dCCNauc02 <- rep(0,k)
dPCCNauc02<- rep(0,k)
signdCCNauc02<- rep(0,k)
signdPCCNauc02<- rep(0,k)
exhaustiveauc02 <- rep(0,k)
dGGMauc02 <- rep(0,k)

################################################
low.ci.dCCNauc <- rep(0,k)
low.ci.dPCCNauc<- rep(0,k)
low.ci.signdCCNauc<- rep(0,k)
low.ci.signdPCCNauc<- rep(0,k)
low.ci.exhaustiveauc <- rep(0,k)
low.ci.dGGMauc <- rep(0,k)

low.ci.dCCNauc02 <- rep(0,k)
low.ci.dPCCNauc02<- rep(0,k)
low.ci.signdCCNauc02<- rep(0,k)
low.ci.signdPCCNauc02<- rep(0,k)
low.ci.exhaustiveauc02 <- rep(0,k)
low.ci.dGGMauc02 <- rep(0,k)
################################################

high.ci.dCCNauc <- rep(0,k)
high.ci.dPCCNauc<- rep(0,k)
high.ci.signdCCNauc<- rep(0,k)
high.ci.signdPCCNauc<- rep(0,k)
high.ci.exhaustiveauc <- rep(0,k)
high.ci.dGGMauc <- rep(0,k)

high.ci.dCCNauc02 <- rep(0,k)
high.ci.dPCCNauc02<- rep(0,k)
high.ci.signdCCNauc02<- rep(0,k)
high.ci.signdPCCNauc02<- rep(0,k)
high.ci.exhaustiveauc02 <- rep(0,k)
high.ci.dGGMauc02 <- rep(0,k)

####################################################


#Indicate the true positives


ROCmatrix <- matrix(0,ncol(rdata),ncol(rdata))

ROCmatrix[75,150] <- 1
ROCmatrix[100,200] <- 1
ROCmatrix[125,215] <- 1
ROCmatrix[25,52] <- 1

ROCmatrix[33,66] <- 1
ROCmatrix[88,144] <- 1

ROCmatrix[2,170] <- 1
ROCmatrix[50,115] <- 1
ROCmatrix[44,99] <- 1

ROCmatrix[12,180] <- 1
ROCmatrix[60,125] <- 1
ROCmatrix[22,190] <- 1
ROCmatrix[22,211] <- 1


ROCmatrix[70,135] <- 1
ROCmatrix[32,221] <- 1
ROCmatrix[12,183] <- 1
ROCmatrix[54,109] <- 1

#True positive for the exhaustive search

observations <-read.csv("~/trainingData-release.csv", header = FALSE)
  observations <- observations[-1,c(42:272)]
  nams <- apply( combn(colnames(observations),2), 2, function(z) paste(z, collapse = '*'))
  cols <- combn(ncol(rdata), 2)
  three <- apply(cols, 2, function(z) rowProds(as.matrix(rdata)[,z]))
  colnames(three) <- nams
  

ROCvec <- rep(0,dim(three)[2])
cvCoefficients <- matrix(0,dim(three)[2],k)
ROCvec[which(colnames(three) == "V116*V191")] <- 1
ROCvec[which(colnames(three) == "V141*V241")] <- 1
ROCvec[which(colnames(three) == "V166*V256")] <- 1
ROCvec[which(colnames(three) == "V66*V93")] <- 1


ROCvec[which(colnames(three) == "V74*V107")] <- 1
ROCvec[which(colnames(three) == "V129*V185")] <- 1

ROCvec[which(colnames(three) == "V43*V211")] <- 1
ROCvec[which(colnames(three) == "V91*V156")] <- 1
ROCvec[which(colnames(three) == "V85*V140")] <- 1

ROCvec[which(colnames(three) == "V53*V221")] <- 1
ROCvec[which(colnames(three) == "V101*V166")] <- 1
ROCvec[which(colnames(three) == "V63*V231")] <- 1
ROCvec[which(colnames(three) == "V63*V252")] <- 1


ROCvec[which(colnames(three) == "V111*V176")] <- 1
ROCvec[which(colnames(three) == "V73*V262")] <- 1
ROCvec[which(colnames(three) == "V53*V224")] <- 1
ROCvec[which(colnames(three) == "V95*V150")] <- 1
```


#SIMULATE THE MODELS AND PERFORM ALL ANALYSES


```
#TWO RELU TERMS

z1 <- rdata[,12]*rdata[,183]
z2 <- rdata[,109]*rdata[,54]

z1[which(z1 < quantile(z1, 0.75))] <-0
z2[which(z2 < quantile(z2, 0.75))] <-0

ymat <- matrix(0,191,k)
for(l in 1:k){
  
  data <-read.csv("~/trainingData-release.csv", header = TRUE)
  rdata <- data[,c(42:272)]
  
  rdata[,125] <- rdata[,215]  + rnorm(191,0,0.25)
  rdata[,75] <- rdata[,150] + rnorm(191,0,0.25)
  
  
  ymat[,l] <- rdata[,75]*rdata[,150]+rdata[,100]*rdata[,200] +rdata[,125]*rdata[,215] +rdata[,25]*rdata[,52]+rdata[,33]*rdata[,66]+rdata[,88]*rdata[,144]+
    2*(z1+z2)+rnorm(191,0,1.75)
  y1 <- ymat[,l]
  her1[l] <- (var(y1)-1.75^2)/var(y1)
  
  rdata[which(y1 < quantile(y1, 0.80)),2] <- -rdata[which(y1 < quantile(y1, 0.80)),170] + rnorm(length(which(y1 < quantile(y1, 0.80))),0,0.25)
  rdata[which(y1 < quantile(y1, 0.80)),50] <- -rdata[which(y1 < quantile(y1, 0.80)),115] + rnorm(length(which(y1 < quantile(y1, 0.80))),0,0.25)
  rdata[which(y1 > quantile(y1, 0.80)),99] <- -rdata[which(y1 > quantile(y1, 0.80)),44] + rnorm(length(which(y1 > quantile(y1, 0.80))),0,0.25)
  
  rdata[which(y1 < quantile(y1, 0.80)),12] <- rdata[which(y1 < quantile(y1, 0.80)),180] + rnorm(length(which(y1 < quantile(y1, 0.80))),0,0.25)
  rdata[which(y1 < quantile(y1, 0.80)),60] <- rdata[which(y1 < quantile(y1, 0.80)),125] + rnorm(length(which(y1 < quantile(y1, 0.80))),0,0.25)
  rdata[which(y1 < quantile(y1, 0.80)),22] <- rdata[which(y1 < quantile(y1, 0.80)),211] + rnorm(length(which(y1 < quantile(y1, 0.80))),0,0.25)
  
  rdata[which(y1 < quantile(y1, 0.80)),22] <- rdata[which(y1 < quantile(y1, 0.80)),190] + rnorm(length(which(y1 < quantile(y1, 0.80))),0,0.25)
  rdata[which(y1 < quantile(y1, 0.80)),70] <- rdata[which(y1 < quantile(y1, 0.80)),135] + rnorm(length(which(y1 < quantile(y1, 0.80))),0,0.25)
  rdata[which(y1 < quantile(y1, 0.80)),32] <- rdata[which(y1 < quantile(y1, 0.80)),221] + rnorm(length(which(y1 < quantile(y1, 0.80))),0,0.25)
  
  data <- rdata
  rdatareps[[l]] <- rdata 
  
  
  #####EXHAUSTIVE SEARCH########################
  
  #Enumerate all possible pairwise interactions
  
  observations <-read.csv("rr.csv", header = FALSE)
  observations <- observations[-1,c(42:272)]
  nams <- apply( combn(colnames(observations),2), 2, function(z) paste(z, collapse = '*'))
  cols <- combn(ncol(rdata), 2)
  three <- apply(cols, 2, function(z) rowProds(as.matrix(rdata)[,z]))
  colnames(three) <- nams
  
  cv.fit=cv.glmnet(three, y1, alpha = 0, maxit = 10000)
  fit=glmnet(three,y1, alpha = 0, maxit = 10000)
  cvCoefficients[,l] <- coef(fit, s = cv.fit$lambda.min)[-1]
  
  
  
  
  
  
  
  ############differential GGM#########
  high <- rdata[which(y1 > quantile(y1, 0.5)),]
  low <- rdata[which(y1 < quantile(y1, 0.5)),]
  fgl.results = JGL(Y=list(low, high),penalty="fused",lambda1=.1,lambda2=.1,return.whole.theta=TRUE)
  GGM <-abs(as.matrix(fgl.results$theta[[1]])-as.matrix(fgl.results$theta[[2]]))
  
  
  ########################
  ###differential DCCN####
  dCCN <- abs(cor(high) - cor(low))
  signdCCN <- (abs(sign(cor(high))-sign(cor(low))))*dCCN
  
  ########################
  
  
  
  
  
  
  ############Sign-adjusted dPCCN#################
  
  #Residual step
  
  rdata <- as.matrix(rdata)
  cv.fit <- cv.glmnet(rdata, scale(y1), alpha = 1.0, maxit = 10000)
  fit=glmnet(rdata, scale(y1), alpha = 1.0, maxit = 10000)
  Coefficients <- coef(fit, s = cv.fit$lambda.min)
  v <- rdata %*% Coefficients[-1]
  resid <- scale(y1)-v
  
  
  #Estimate dPCCN structures
  #The threshold a is used to define whether or not a estimated matrix element is zero (See the "Methods" section).
  a = 0.1
  dPCCN <- matrix(0,ncol(data),ncol(data))
  signP <- matrix(0,ncol(data),ncol(data))
  
  
  for(i in 1:ncol(data)){
    for(j in 1:ncol(data)){
      
      res <- (lm(rdata[,j]~rdata[,i])$residuals)
      set <- cbind(resid,res,rdata[,i])
      high <- set[which(resid > quantile(resid, 1/2)),]
      low <- set[which(resid < quantile(resid,1/2)),]
      high <- high[,-1]
      low <- low[,-1]
      
      
      if(abs(cor(high[,1],high[,2])) < a){
        h <- 0
      }else{
        h <- cor(high[,1],high[,2])
      }
      
      if(abs(cor(low[,1],low[,2])) < a){
        L <- 0
      }else{
        L <- cor(low[,1],low[,2])
      }
      
      dPCCN[i,j] <- h-L
      
      signP[i,j] <- 0.5*abs((sign(h)-sign(L)))
      
      
    }
  }
  
  diag(dPCCN) <- 0
  
  signP[signP != 0 ] <- 1
  signdPCCN <- abs(signP*dPCCN)
  
  
  
  
  #Calculate AUCS for each replication
  
  dCCNauc02[l] <- roc(ROCmatrix[upper.tri(ROCmatrix)],dCCN[upper.tri(dCCN)], partial.auc = c(0.8,1.0), partial.auc.correct = TRUE)$auc
  dPCCNauc02[l]<- roc(ROCmatrix[upper.tri(ROCmatrix)],dPCCN[upper.tri(dPCCN)], partial.auc = c(0.8,1.0), partial.auc.correct = TRUE)$auc
  signdCCNauc02[l]<- roc(ROCmatrix[upper.tri(ROCmatrix)],signdCCN[upper.tri(signdCCN)], partial.auc = c(0.8,1.0), partial.auc.correct = TRUE)$auc
  signdPCCNauc02[l]<- roc(ROCmatrix[upper.tri(ROCmatrix)],signdPCCN[upper.tri(signdPCCN)], partial.auc = c(0.8,1.0), partial.auc.correct = TRUE)$auc
  exhaustiveauc02[l] <- roc(ROCvec,abs(cvCoefficients[,l]),partial.auc = c(0.8,1.0), partial.auc.correct = TRUE)$auc
  
  dCCNauc[l] <- roc(ROCmatrix[upper.tri(ROCmatrix)],dCCN[upper.tri(dCCN)])$auc
  dPCCNauc[l]<- roc(ROCmatrix[upper.tri(ROCmatrix)],dPCCN[upper.tri(dPCCN)])$auc
  signdCCNauc[l]<- roc(ROCmatrix[upper.tri(ROCmatrix)],signdCCN[upper.tri(signdCCN)])$auc
  signdPCCNauc[l]<- roc(ROCmatrix[upper.tri(ROCmatrix)],signdPCCN[upper.tri(signdPCCN)])$auc
  exhaustiveauc[l] <- roc(ROCvec, abs(cvCoefficients[,l]))$auc
  
  
  dGGMauc[l] <-  roc(ROCmatrix[upper.tri(ROCmatrix)],GGM[upper.tri(as.matrix(GGM))])$auc
  dGGMauc02[l] <-  roc(ROCmatrix[upper.tri(ROCmatrix)],GGM[upper.tri(as.matrix(GGM))], partial.auc = c(0.8,1.0), partial.auc.correct = TRUE)$auc
  
  
  
  ########################################
  
  
  low.ci.dCCNauc02[l] <- ci.auc(roc(ROCmatrix[upper.tri(ROCmatrix)],dCCN[upper.tri(dCCN)], partial.auc = c(0.8,1.0), partial.auc.correct = TRUE)$auc)[1]
  low.ci.dPCCNauc02[l]<- ci.auc(roc(ROCmatrix[upper.tri(ROCmatrix)],dPCCN[upper.tri(dPCCN)], partial.auc = c(0.8,1.0), partial.auc.correct = TRUE)$auc)[1]
  low.ci.signdCCNauc02[l]<- ci.auc(roc(ROCmatrix[upper.tri(ROCmatrix)],signdCCN[upper.tri(signdCCN)], partial.auc = c(0.8,1.0), partial.auc.correct = TRUE)$auc)[1]
  low.ci.signdPCCNauc02[l]<- ci.auc(roc(ROCmatrix[upper.tri(ROCmatrix)],signdPCCN[upper.tri(signdPCCN)], partial.auc = c(0.8,1.0), partial.auc.correct = TRUE)$auc)[1]
  low.ci.exhaustiveauc02[l] <- ci.auc(roc(ROCvec,abs(cvCoefficients[,l]),partial.auc = c(0.8,1.0), partial.auc.correct = TRUE)$auc)[1]
  
  low.ci.dCCNauc[l] <- ci.auc(roc(ROCmatrix[upper.tri(ROCmatrix)],dCCN[upper.tri(dCCN)])$auc)[1]
  low.ci.dPCCNauc[l]<- ci.auc(roc(ROCmatrix[upper.tri(ROCmatrix)],dPCCN[upper.tri(dPCCN)])$auc)[1]
  low.ci.signdCCNauc[l]<- ci.auc(roc(ROCmatrix[upper.tri(ROCmatrix)],signdCCN[upper.tri(signdCCN)])$auc)[1]
  low.ci.signdPCCNauc[l]<- ci.auc(roc(ROCmatrix[upper.tri(ROCmatrix)],signdPCCN[upper.tri(signdPCCN)])$auc)[1]
  low.ci.exhaustiveauc[l] <- ci.auc(roc(ROCvec, abs(cvCoefficients[,l]))$auc)[1]
  
  
  low.ci.dGGMauc[l] <-  ci.auc(roc(ROCmatrix[upper.tri(ROCmatrix)],GGM[upper.tri(as.matrix(GGM))])$auc)[1]
  low.ci.dGGMauc02[l] <-  ci.auc(roc(ROCmatrix[upper.tri(ROCmatrix)],GGM[upper.tri(as.matrix(GGM))], partial.auc = c(0.8,1.0), partial.auc.correct = TRUE)$auc)[1]
  
  
  #######################################################
  
  high.ci.dCCNauc02[l] <- ci.auc(roc(ROCmatrix[upper.tri(ROCmatrix)],dCCN[upper.tri(dCCN)], partial.auc = c(0.8,1.0), partial.auc.correct = TRUE)$auc)[3]
  high.ci.dPCCNauc02[l]<- ci.auc(roc(ROCmatrix[upper.tri(ROCmatrix)],dPCCN[upper.tri(dPCCN)], partial.auc = c(0.8,1.0), partial.auc.correct = TRUE)$auc)[3]
  high.ci.signdCCNauc02[l]<- ci.auc(roc(ROCmatrix[upper.tri(ROCmatrix)],signdCCN[upper.tri(signdCCN)], partial.auc = c(0.8,1.0), partial.auc.correct = TRUE)$auc)[3]
  high.ci.signdPCCNauc02[l]<- ci.auc(roc(ROCmatrix[upper.tri(ROCmatrix)],signdPCCN[upper.tri(signdPCCN)], partial.auc = c(0.8,1.0), partial.auc.correct = TRUE)$auc)[3]
  high.ci.exhaustiveauc02[l] <- ci.auc(roc(ROCvec,abs(cvCoefficients[,l]),partial.auc = c(0.8,1.0), partial.auc.correct = TRUE)$auc)[3]
  
  high.ci.dCCNauc[l] <- ci.auc(roc(ROCmatrix[upper.tri(ROCmatrix)],dCCN[upper.tri(dCCN)])$auc)[3]
  high.ci.dPCCNauc[l]<- ci.auc(roc(ROCmatrix[upper.tri(ROCmatrix)],dPCCN[upper.tri(dPCCN)])$auc)[3]
  high.ci.signdCCNauc[l]<- ci.auc(roc(ROCmatrix[upper.tri(ROCmatrix)],signdCCN[upper.tri(signdCCN)])$auc)[3]
  high.ci.signdPCCNauc[l]<- ci.auc(roc(ROCmatrix[upper.tri(ROCmatrix)],signdPCCN[upper.tri(signdPCCN)])$auc)[3]
  high.ci.exhaustiveauc[l] <- ci.auc(roc(ROCvec, abs(cvCoefficients[,l]))$auc)[3]
  
  
  high.ci.dGGMauc[l] <-  ci.auc(roc(ROCmatrix[upper.tri(ROCmatrix)],GGM[upper.tri(as.matrix(GGM))])$auc)[3]
  high.ci.dGGMauc02[l] <-  ci.auc(roc(ROCmatrix[upper.tri(ROCmatrix)],GGM[upper.tri(as.matrix(GGM))], partial.auc = c(0.8,1.0), partial.auc.correct = TRUE)$auc)[3]
  
  }
```


#AVERAGE THE RESULTS OVER REPLICATIONS


```
c(mean(dCCNauc02), mean(low.ci.dCCNauc02),mean(high.ci.dCCNauc02))
c(mean(signdCCNauc02,na.rm=TRUE), mean(low.ci.signdCCNauc02),mean(high.ci.signdCCNauc02))
c(mean(signdPCCNauc02), mean(low.ci.signdPCCNauc02),mean(high.ci.signdPCCNauc02))
c(mean(exhaustiveauc02,na.rm=TRUE), mean(low.ci.exhaustiveauc02),mean(high.ci.exhaustiveauc02))
c(mean(dGGMauc02), mean(low.ci.dGGMauc02),mean(high.ci.dGGMauc02))


c(mean(dCCNauc), mean(low.ci.dCCNauc),mean(high.ci.dCCNauc))
c(mean(signdCCNauc), mean(low.ci.signdCCNauc),mean(high.ci.signdCCNauc))
c(mean(signdPCCNauc), mean(low.ci.signdPCCNauc),mean(high.ci.signdPCCNauc))
c(mean(exhaustiveauc), mean(low.ci.exhaustiveauc),mean(high.ci.exhaustiveauc))
c(mean(dGGMauc), mean(low.ci.dGGMauc),mean(high.ci.dGGMauc))
```


LS0tDQp0aXRsZTogIlNJTVVMQVRFRCBFWEFNUExFUzogTU9ERUwgQSINCm91dHB1dDogaHRtbF9ub3RlYm9vaw0KLS0tDQoNCkdvIHRvIGEgd2Vic2l0ZSAoaHR0cHM6Ly93d3cuc3luYXBzZS5vcmcvIyFTeW5hcHNlOnN5bjI0NTU2ODMvd2lraS82NDAwNykgYW5kIGRvd25sb2FkIGEgbm9ybWFsaXplZCBwcm90ZWluIGV4cHJlc3Npb24gRFJFQU05LWNoYWxsZW5nZSBkYXRhc2V0ICh0cmFpbmluZ0RhdGEtcmVsZWFzZS5jc3YpIChOb3JlbiBldCBhbC4yMDE2IC0gaHR0cHM6Ly9kb2kub3JnLzEwLjEzNzEvam91cm5hbC5wY2JpLjEwMDQ4OTApIHdoaWNoIGlzIGF2YWlsYWJsZSB1cG9uIHJlZ2lzdHJhdGlvbiAoaHR0cDovL2RyZWFtY2hhbGxlbmdlcy5vcmcvKS4gVGhlc2UgZGF0YSB3ZXJlIHByb3ZpZGVkIGJ5IERyLiBTdGV2ZW4gS29ybmJsYXUgZnJvbSB0aGUgVW5pdmVyc2l0eSBvZiBUZXhhcyBNRCBBbmRlcnNvbiBDYW5jZXIgQ2VudGVyIGFuZCB3ZXJlIG9idGFpbmVkIHRocm91Z2ggU3luYXBzZSBzeW4yNDU1NjgzIGFzIGEgcGFydCBvZiB0aGUgYWN1dGUgbXllbG9pZCBsZXVrZW1pYSAoQU1MKSBEUkVBTS1jaGFsbGVuZ2UuIA0KDQojU1RFUCAwOiBQUkVQQVJJTkcgVEhFIERBVEFTRVQgQU5EIElOU1RBTExJTkcgVEhFIFJFUVVJUkVEIFItUEFDS0FHRVMNCkluc3RhbGwgdGhlIHJlcXVpcmVkIHBhY2thZ2VzIGFuZCBwcmVwYXJlIHRoZSBBTUwgZGF0YXNldCBmcm9tIHRoZSBkb3dubG9hZGVkIENTVi1maWxlICJ0cmFpbmluZ0RhdGEtcmVsZWFzZS5jc3YiIChyZXF1aXJlcyByZWdpc3RyYXRpb24pIC0gaHR0cHM6Ly93d3cuc3luYXBzZS5vcmcvIyFTeW5hcHNlOnN5bjI0NTU2ODMvd2lraS82NDAwNy4NCg0KYGBge3J9DQojRE9XTkxPQUQgUkVRVUlSRUQgUi1QQUNLQUdFUw0KbGlicmFyeSgiZ2xtbmV0IikgI2h0dHBzOi8vQ1JBTi5SLXByb2plY3Qub3JnL3BhY2thZ2U9Z2xtbmV0DQpsaWJyYXJ5KCJtYXRyaXhTdGF0cyIpICNodHRwczovL0NSQU4uUi1wcm9qZWN0Lm9yZy9wYWNrYWdlPW1hdHJpeFN0YXRzDQpsaWJyYXJ5KCJxZ3JhcGgiKSAjaHR0cHM6Ly9DUkFOLlItcHJvamVjdC5vcmcvcGFja2FnZT1xZ3JhcGggDQpsaWJyYXJ5KCJuZXRkaWZmdXNlUiIpICNodHRwczovL0NSQU4uUi1wcm9qZWN0Lm9yZy9wYWNrYWdlPW5ldGRpZmZ1c2VSDQpsaWJyYXJ5KCJzdHJpbmdyIikgI2h0dHBzOi8vQ1JBTi5SLXByb2plY3Qub3JnL3BhY2thZ2U9c3RyaW5nciANCg0KI1JlcGxhY2UgIn4vdHJhaW5pbmdEYXRhLXJlbGVhc2UuY3N2IiB3aXRoIGEgdXNlci1zcGVjaWZpYyBwYXRoIHRvIHRoZSBkb3dubG9hZGVkIENTVi1maWxlLg0KZGF0YSA8LXJlYWQuY3N2KCJ+L3RyYWluaW5nRGF0YS1yZWxlYXNlLmNzdiIsIGhlYWRlciA9IFRSVUUpDQoNCiNTZXBhcmF0ZSB0aGUgZXhwcmVzc2lvbiBkYXRhIChyZW5hbWVkIGFzICJyZGF0YSIpIGZyb20gY2xpbmljYWwgY292YXJpYXRlcy4NCnJkYXRhIDwtIGRhdGFbLGMoNDI6MjcyKV0NCg0KYGBgDQojSU5JVElBTElaRQ0KYGBge3J9DQoNCiNOdW1iZXIgb2YgcmVwbGljYXRlcw0KayA9IDEwDQpkYXRhIDwtIHJkYXRhDQojRW1wdHkgbGlzdCBhbmQgdmVjdG9ycyBmb3IgcmVwbGljYXRlcw0KZENDTmxpc3QgPC0gcmVwKGxpc3QobWF0cml4KE5BLCBuY29sKHJkYXRhKSwgbmNvbChyZGF0YSkpLCBrKSkNCmRQQ0NObGlzdCA8LSByZXAobGlzdChtYXRyaXgoTkEsIG5jb2wocmRhdGEpLCBuY29sKHJkYXRhKSksIGspKQ0KR0dNbGlzdCA8LSByZXAobGlzdChtYXRyaXgoTkEsIG5jb2wocmRhdGEpLCBuY29sKHJkYXRhKSksIGspKQ0KeW1hdCA8LSBtYXRyaXgoMCwxOTEsaykNCmhlcjEgPC0gcmVwKDAsaykNCnJkYXRhcmVwcyA8LSByZXAobGlzdChtYXRyaXgoTkEsIG5jb2wocmRhdGEpLCBuY29sKHJkYXRhKSkpLCBrKQ0KDQojRW1wdHkgdmVjdG9ycyBmb3IgQVVDUyBhbmQgdGhlaXIgY29uZmlkZW5jZSBpbnRlcnZhbHMgY2FsY3VsYXRlZCBvdmVyIHJlcGxpY2F0ZXMNCg0KZENDTmF1YyA8LSByZXAoMCxrKQ0KZFBDQ05hdWM8LSByZXAoMCxrKQ0Kc2lnbmRDQ05hdWM8LSByZXAoMCxrKQ0Kc2lnbmRQQ0NOYXVjPC0gcmVwKDAsaykNCmV4aGF1c3RpdmVhdWMgPC0gcmVwKDAsaykNCmRHR01hdWMgPC0gcmVwKDAsaykNCg0KZENDTmF1YzAyIDwtIHJlcCgwLGspDQpkUENDTmF1YzAyPC0gcmVwKDAsaykNCnNpZ25kQ0NOYXVjMDI8LSByZXAoMCxrKQ0Kc2lnbmRQQ0NOYXVjMDI8LSByZXAoMCxrKQ0KZXhoYXVzdGl2ZWF1YzAyIDwtIHJlcCgwLGspDQpkR0dNYXVjMDIgPC0gcmVwKDAsaykNCg0KIyMjIyMjIyMjIyMjIyMjIyMjIyMjIyMjIyMjIyMjIyMjIyMjIyMjIyMjIyMjIyMjDQpsb3cuY2kuZENDTmF1YyA8LSByZXAoMCxrKQ0KbG93LmNpLmRQQ0NOYXVjPC0gcmVwKDAsaykNCmxvdy5jaS5zaWduZENDTmF1YzwtIHJlcCgwLGspDQpsb3cuY2kuc2lnbmRQQ0NOYXVjPC0gcmVwKDAsaykNCmxvdy5jaS5leGhhdXN0aXZlYXVjIDwtIHJlcCgwLGspDQpsb3cuY2kuZEdHTWF1YyA8LSByZXAoMCxrKQ0KDQpsb3cuY2kuZENDTmF1YzAyIDwtIHJlcCgwLGspDQpsb3cuY2kuZFBDQ05hdWMwMjwtIHJlcCgwLGspDQpsb3cuY2kuc2lnbmRDQ05hdWMwMjwtIHJlcCgwLGspDQpsb3cuY2kuc2lnbmRQQ0NOYXVjMDI8LSByZXAoMCxrKQ0KbG93LmNpLmV4aGF1c3RpdmVhdWMwMiA8LSByZXAoMCxrKQ0KbG93LmNpLmRHR01hdWMwMiA8LSByZXAoMCxrKQ0KIyMjIyMjIyMjIyMjIyMjIyMjIyMjIyMjIyMjIyMjIyMjIyMjIyMjIyMjIyMjIyMjDQoNCmhpZ2guY2kuZENDTmF1YyA8LSByZXAoMCxrKQ0KaGlnaC5jaS5kUENDTmF1YzwtIHJlcCgwLGspDQpoaWdoLmNpLnNpZ25kQ0NOYXVjPC0gcmVwKDAsaykNCmhpZ2guY2kuc2lnbmRQQ0NOYXVjPC0gcmVwKDAsaykNCmhpZ2guY2kuZXhoYXVzdGl2ZWF1YyA8LSByZXAoMCxrKQ0KaGlnaC5jaS5kR0dNYXVjIDwtIHJlcCgwLGspDQoNCmhpZ2guY2kuZENDTmF1YzAyIDwtIHJlcCgwLGspDQpoaWdoLmNpLmRQQ0NOYXVjMDI8LSByZXAoMCxrKQ0KaGlnaC5jaS5zaWduZENDTmF1YzAyPC0gcmVwKDAsaykNCmhpZ2guY2kuc2lnbmRQQ0NOYXVjMDI8LSByZXAoMCxrKQ0KaGlnaC5jaS5leGhhdXN0aXZlYXVjMDIgPC0gcmVwKDAsaykNCmhpZ2guY2kuZEdHTWF1YzAyIDwtIHJlcCgwLGspDQoNCiMjIyMjIyMjIyMjIyMjIyMjIyMjIyMjIyMjIyMjIyMjIyMjIyMjIyMjIyMjIyMjIyMjIyMNCg0KDQojSW5kaWNhdGUgdGhlIHRydWUgcG9zaXRpdmVzDQoNCg0KUk9DbWF0cml4IDwtIG1hdHJpeCgwLG5jb2wocmRhdGEpLG5jb2wocmRhdGEpKQ0KDQpST0NtYXRyaXhbNzUsMTUwXSA8LSAxDQpST0NtYXRyaXhbMTAwLDIwMF0gPC0gMQ0KUk9DbWF0cml4WzEyNSwyMTVdIDwtIDENClJPQ21hdHJpeFsyNSw1Ml0gPC0gMQ0KDQpST0NtYXRyaXhbMzMsNjZdIDwtIDENClJPQ21hdHJpeFs4OCwxNDRdIDwtIDENCg0KUk9DbWF0cml4WzIsMTcwXSA8LSAxDQpST0NtYXRyaXhbNTAsMTE1XSA8LSAxDQpST0NtYXRyaXhbNDQsOTldIDwtIDENCg0KUk9DbWF0cml4WzEyLDE4MF0gPC0gMQ0KUk9DbWF0cml4WzYwLDEyNV0gPC0gMQ0KUk9DbWF0cml4WzIyLDE5MF0gPC0gMQ0KUk9DbWF0cml4WzIyLDIxMV0gPC0gMQ0KDQoNCg0KUk9DbWF0cml4WzcwLDEzNV0gPC0gMQ0KUk9DbWF0cml4WzMyLDIyMV0gPC0gMQ0KUk9DbWF0cml4WzEyLDE4M10gPC0gMQ0KUk9DbWF0cml4WzU0LDEwOV0gPC0gMQ0KDQojVHJ1ZSBwb3NpdGl2ZSBmb3IgdGhlIGV4aGF1c3RpdmUgc2VhcmNoDQoNCm9ic2VydmF0aW9ucyA8LXJlYWQuY3N2KCJ+L3RyYWluaW5nRGF0YS1yZWxlYXNlLmNzdiIsIGhlYWRlciA9IEZBTFNFKQ0KICBvYnNlcnZhdGlvbnMgPC0gb2JzZXJ2YXRpb25zWy0xLGMoNDI6MjcyKV0NCiAgbmFtcyA8LSBhcHBseSggY29tYm4oY29sbmFtZXMob2JzZXJ2YXRpb25zKSwyKSwgMiwgZnVuY3Rpb24oeikgcGFzdGUoeiwgY29sbGFwc2UgPSAnKicpKQ0KICBjb2xzIDwtIGNvbWJuKG5jb2wocmRhdGEpLCAyKQ0KICB0aHJlZSA8LSBhcHBseShjb2xzLCAyLCBmdW5jdGlvbih6KSByb3dQcm9kcyhhcy5tYXRyaXgocmRhdGEpWyx6XSkpDQogIGNvbG5hbWVzKHRocmVlKSA8LSBuYW1zDQogIA0KDQpST0N2ZWMgPC0gcmVwKDAsZGltKHRocmVlKVsyXSkNCmN2Q29lZmZpY2llbnRzIDwtIG1hdHJpeCgwLGRpbSh0aHJlZSlbMl0saykNClJPQ3ZlY1t3aGljaChjb2xuYW1lcyh0aHJlZSkgPT0gIlYxMTYqVjE5MSIpXSA8LSAxDQpST0N2ZWNbd2hpY2goY29sbmFtZXModGhyZWUpID09ICJWMTQxKlYyNDEiKV0gPC0gMQ0KUk9DdmVjW3doaWNoKGNvbG5hbWVzKHRocmVlKSA9PSAiVjE2NipWMjU2IildIDwtIDENClJPQ3ZlY1t3aGljaChjb2xuYW1lcyh0aHJlZSkgPT0gIlY2NipWOTMiKV0gPC0gMQ0KDQoNClJPQ3ZlY1t3aGljaChjb2xuYW1lcyh0aHJlZSkgPT0gIlY3NCpWMTA3IildIDwtIDENClJPQ3ZlY1t3aGljaChjb2xuYW1lcyh0aHJlZSkgPT0gIlYxMjkqVjE4NSIpXSA8LSAxDQoNClJPQ3ZlY1t3aGljaChjb2xuYW1lcyh0aHJlZSkgPT0gIlY0MypWMjExIildIDwtIDENClJPQ3ZlY1t3aGljaChjb2xuYW1lcyh0aHJlZSkgPT0gIlY5MSpWMTU2IildIDwtIDENClJPQ3ZlY1t3aGljaChjb2xuYW1lcyh0aHJlZSkgPT0gIlY4NSpWMTQwIildIDwtIDENCg0KUk9DdmVjW3doaWNoKGNvbG5hbWVzKHRocmVlKSA9PSAiVjUzKlYyMjEiKV0gPC0gMQ0KUk9DdmVjW3doaWNoKGNvbG5hbWVzKHRocmVlKSA9PSAiVjEwMSpWMTY2IildIDwtIDENClJPQ3ZlY1t3aGljaChjb2xuYW1lcyh0aHJlZSkgPT0gIlY2MypWMjMxIildIDwtIDENClJPQ3ZlY1t3aGljaChjb2xuYW1lcyh0aHJlZSkgPT0gIlY2MypWMjUyIildIDwtIDENCg0KDQoNClJPQ3ZlY1t3aGljaChjb2xuYW1lcyh0aHJlZSkgPT0gIlYxMTEqVjE3NiIpXSA8LSAxDQpST0N2ZWNbd2hpY2goY29sbmFtZXModGhyZWUpID09ICJWNzMqVjI2MiIpXSA8LSAxDQpST0N2ZWNbd2hpY2goY29sbmFtZXModGhyZWUpID09ICJWNTMqVjIyNCIpXSA8LSAxDQpST0N2ZWNbd2hpY2goY29sbmFtZXModGhyZWUpID09ICJWOTUqVjE1MCIpXSA8LSAxDQpgYGANCiNTSU1VTEFURSBUSEUgTU9ERUxTIEFORCBQRVJGT1JNIEFMTCBBTkFMWVNFUw0KYGBge3J9DQoNCiNUV08gUkVMVSBURVJNUw0KDQp6MSA8LSByZGF0YVssMTJdKnJkYXRhWywxODNdDQp6MiA8LSByZGF0YVssMTA5XSpyZGF0YVssNTRdDQoNCnoxW3doaWNoKHoxIDwgcXVhbnRpbGUoejEsIDAuNzUpKV0gPC0wDQp6Mlt3aGljaCh6MiA8IHF1YW50aWxlKHoyLCAwLjc1KSldIDwtMA0KDQp5bWF0IDwtIG1hdHJpeCgwLDE5MSxrKQ0KZm9yKGwgaW4gMTprKXsNCiAgDQogIGRhdGEgPC1yZWFkLmNzdigifi90cmFpbmluZ0RhdGEtcmVsZWFzZS5jc3YiLCBoZWFkZXIgPSBUUlVFKQ0KICByZGF0YSA8LSBkYXRhWyxjKDQyOjI3MildDQogIA0KICByZGF0YVssMTI1XSA8LSByZGF0YVssMjE1XSAgKyBybm9ybSgxOTEsMCwwLjI1KQ0KICByZGF0YVssNzVdIDwtIHJkYXRhWywxNTBdICsgcm5vcm0oMTkxLDAsMC4yNSkNCiAgDQogIA0KICB5bWF0WyxsXSA8LSByZGF0YVssNzVdKnJkYXRhWywxNTBdK3JkYXRhWywxMDBdKnJkYXRhWywyMDBdICtyZGF0YVssMTI1XSpyZGF0YVssMjE1XSArcmRhdGFbLDI1XSpyZGF0YVssNTJdK3JkYXRhWywzM10qcmRhdGFbLDY2XStyZGF0YVssODhdKnJkYXRhWywxNDRdKw0KICAgIDIqKHoxK3oyKStybm9ybSgxOTEsMCwxLjc1KQ0KICB5MSA8LSB5bWF0WyxsXQ0KICBoZXIxW2xdIDwtICh2YXIoeTEpLTEuNzVeMikvdmFyKHkxKQ0KICANCiAgcmRhdGFbd2hpY2goeTEgPCBxdWFudGlsZSh5MSwgMC44MCkpLDJdIDwtIC1yZGF0YVt3aGljaCh5MSA8IHF1YW50aWxlKHkxLCAwLjgwKSksMTcwXSArIHJub3JtKGxlbmd0aCh3aGljaCh5MSA8IHF1YW50aWxlKHkxLCAwLjgwKSkpLDAsMC4yNSkNCiAgcmRhdGFbd2hpY2goeTEgPCBxdWFudGlsZSh5MSwgMC44MCkpLDUwXSA8LSAtcmRhdGFbd2hpY2goeTEgPCBxdWFudGlsZSh5MSwgMC44MCkpLDExNV0gKyBybm9ybShsZW5ndGgod2hpY2goeTEgPCBxdWFudGlsZSh5MSwgMC44MCkpKSwwLDAuMjUpDQogIHJkYXRhW3doaWNoKHkxID4gcXVhbnRpbGUoeTEsIDAuODApKSw5OV0gPC0gLXJkYXRhW3doaWNoKHkxID4gcXVhbnRpbGUoeTEsIDAuODApKSw0NF0gKyBybm9ybShsZW5ndGgod2hpY2goeTEgPiBxdWFudGlsZSh5MSwgMC44MCkpKSwwLDAuMjUpDQogIA0KICByZGF0YVt3aGljaCh5MSA8IHF1YW50aWxlKHkxLCAwLjgwKSksMTJdIDwtIHJkYXRhW3doaWNoKHkxIDwgcXVhbnRpbGUoeTEsIDAuODApKSwxODBdICsgcm5vcm0obGVuZ3RoKHdoaWNoKHkxIDwgcXVhbnRpbGUoeTEsIDAuODApKSksMCwwLjI1KQ0KICByZGF0YVt3aGljaCh5MSA8IHF1YW50aWxlKHkxLCAwLjgwKSksNjBdIDwtIHJkYXRhW3doaWNoKHkxIDwgcXVhbnRpbGUoeTEsIDAuODApKSwxMjVdICsgcm5vcm0obGVuZ3RoKHdoaWNoKHkxIDwgcXVhbnRpbGUoeTEsIDAuODApKSksMCwwLjI1KQ0KICByZGF0YVt3aGljaCh5MSA8IHF1YW50aWxlKHkxLCAwLjgwKSksMjJdIDwtIHJkYXRhW3doaWNoKHkxIDwgcXVhbnRpbGUoeTEsIDAuODApKSwyMTFdICsgcm5vcm0obGVuZ3RoKHdoaWNoKHkxIDwgcXVhbnRpbGUoeTEsIDAuODApKSksMCwwLjI1KQ0KICANCiAgcmRhdGFbd2hpY2goeTEgPCBxdWFudGlsZSh5MSwgMC44MCkpLDIyXSA8LSByZGF0YVt3aGljaCh5MSA8IHF1YW50aWxlKHkxLCAwLjgwKSksMTkwXSArIHJub3JtKGxlbmd0aCh3aGljaCh5MSA8IHF1YW50aWxlKHkxLCAwLjgwKSkpLDAsMC4yNSkNCiAgcmRhdGFbd2hpY2goeTEgPCBxdWFudGlsZSh5MSwgMC44MCkpLDcwXSA8LSByZGF0YVt3aGljaCh5MSA8IHF1YW50aWxlKHkxLCAwLjgwKSksMTM1XSArIHJub3JtKGxlbmd0aCh3aGljaCh5MSA8IHF1YW50aWxlKHkxLCAwLjgwKSkpLDAsMC4yNSkNCiAgcmRhdGFbd2hpY2goeTEgPCBxdWFudGlsZSh5MSwgMC44MCkpLDMyXSA8LSByZGF0YVt3aGljaCh5MSA8IHF1YW50aWxlKHkxLCAwLjgwKSksMjIxXSArIHJub3JtKGxlbmd0aCh3aGljaCh5MSA8IHF1YW50aWxlKHkxLCAwLjgwKSkpLDAsMC4yNSkNCiAgDQogIGRhdGEgPC0gcmRhdGENCiAgcmRhdGFyZXBzW1tsXV0gPC0gcmRhdGEgDQogIA0KICANCiAgIyMjIyNFWEhBVVNUSVZFIFNFQVJDSCMjIyMjIyMjIyMjIyMjIyMjIyMjIyMjIw0KICANCiAgI0VudW1lcmF0ZSBhbGwgcG9zc2libGUgcGFpcndpc2UgaW50ZXJhY3Rpb25zDQogIA0KICBvYnNlcnZhdGlvbnMgPC1yZWFkLmNzdigicnIuY3N2IiwgaGVhZGVyID0gRkFMU0UpDQogIG9ic2VydmF0aW9ucyA8LSBvYnNlcnZhdGlvbnNbLTEsYyg0MjoyNzIpXQ0KICBuYW1zIDwtIGFwcGx5KCBjb21ibihjb2xuYW1lcyhvYnNlcnZhdGlvbnMpLDIpLCAyLCBmdW5jdGlvbih6KSBwYXN0ZSh6LCBjb2xsYXBzZSA9ICcqJykpDQogIGNvbHMgPC0gY29tYm4obmNvbChyZGF0YSksIDIpDQogIHRocmVlIDwtIGFwcGx5KGNvbHMsIDIsIGZ1bmN0aW9uKHopIHJvd1Byb2RzKGFzLm1hdHJpeChyZGF0YSlbLHpdKSkNCiAgY29sbmFtZXModGhyZWUpIDwtIG5hbXMNCiAgDQogIGN2LmZpdD1jdi5nbG1uZXQodGhyZWUsIHkxLCBhbHBoYSA9IDAsIG1heGl0ID0gMTAwMDApDQogIGZpdD1nbG1uZXQodGhyZWUseTEsIGFscGhhID0gMCwgbWF4aXQgPSAxMDAwMCkNCiAgY3ZDb2VmZmljaWVudHNbLGxdIDwtIGNvZWYoZml0LCBzID0gY3YuZml0JGxhbWJkYS5taW4pWy0xXQ0KICANCiAgDQogIA0KICANCiAgDQogIA0KICANCiAgIyMjIyMjIyMjIyMjZGlmZmVyZW50aWFsIEdHTSMjIyMjIyMjIw0KICBoaWdoIDwtIHJkYXRhW3doaWNoKHkxID4gcXVhbnRpbGUoeTEsIDAuNSkpLF0NCiAgbG93IDwtIHJkYXRhW3doaWNoKHkxIDwgcXVhbnRpbGUoeTEsIDAuNSkpLF0NCiAgZmdsLnJlc3VsdHMgPSBKR0woWT1saXN0KGxvdywgaGlnaCkscGVuYWx0eT0iZnVzZWQiLGxhbWJkYTE9LjEsbGFtYmRhMj0uMSxyZXR1cm4ud2hvbGUudGhldGE9VFJVRSkNCiAgR0dNIDwtYWJzKGFzLm1hdHJpeChmZ2wucmVzdWx0cyR0aGV0YVtbMV1dKS1hcy5tYXRyaXgoZmdsLnJlc3VsdHMkdGhldGFbWzJdXSkpDQogIA0KICANCiAgIyMjIyMjIyMjIyMjIyMjIyMjIyMjIyMjDQogICMjI2RpZmZlcmVudGlhbCBEQ0NOIyMjIw0KICBkQ0NOIDwtIGFicyhjb3IoaGlnaCkgLSBjb3IobG93KSkNCiAgc2lnbmRDQ04gPC0gKGFicyhzaWduKGNvcihoaWdoKSktc2lnbihjb3IobG93KSkpKSpkQ0NODQogIA0KICAjIyMjIyMjIyMjIyMjIyMjIyMjIyMjIyMNCiAgDQogIA0KICANCiAgDQogIA0KICANCiAgIyMjIyMjIyMjIyMjU2lnbi1hZGp1c3RlZCBkUENDTiMjIyMjIyMjIyMjIyMjIyMjDQogIA0KICAjUmVzaWR1YWwgc3RlcA0KICANCiAgcmRhdGEgPC0gYXMubWF0cml4KHJkYXRhKQ0KICBjdi5maXQgPC0gY3YuZ2xtbmV0KHJkYXRhLCBzY2FsZSh5MSksIGFscGhhID0gMS4wLCBtYXhpdCA9IDEwMDAwKQ0KICBmaXQ9Z2xtbmV0KHJkYXRhLCBzY2FsZSh5MSksIGFscGhhID0gMS4wLCBtYXhpdCA9IDEwMDAwKQ0KICBDb2VmZmljaWVudHMgPC0gY29lZihmaXQsIHMgPSBjdi5maXQkbGFtYmRhLm1pbikNCiAgdiA8LSByZGF0YSAlKiUgQ29lZmZpY2llbnRzWy0xXQ0KICByZXNpZCA8LSBzY2FsZSh5MSktdg0KICANCiAgDQogICNFc3RpbWF0ZSBkUENDTiBzdHJ1Y3R1cmVzDQogICNUaGUgdGhyZXNob2xkIGEgaXMgdXNlZCB0byBkZWZpbmUgd2hldGhlciBvciBub3QgYSBlc3RpbWF0ZWQgbWF0cml4IGVsZW1lbnQgaXMgemVybyAoU2VlIHRoZSAiTWV0aG9kcyIgc2VjdGlvbikuDQogIGEgPSAwLjENCiAgZFBDQ04gPC0gbWF0cml4KDAsbmNvbChkYXRhKSxuY29sKGRhdGEpKQ0KICBzaWduUCA8LSBtYXRyaXgoMCxuY29sKGRhdGEpLG5jb2woZGF0YSkpDQogIA0KICANCiAgZm9yKGkgaW4gMTpuY29sKGRhdGEpKXsNCiAgICBmb3IoaiBpbiAxOm5jb2woZGF0YSkpew0KICAgICAgDQogICAgICByZXMgPC0gKGxtKHJkYXRhWyxqXX5yZGF0YVssaV0pJHJlc2lkdWFscykNCiAgICAgIHNldCA8LSBjYmluZChyZXNpZCxyZXMscmRhdGFbLGldKQ0KICAgICAgaGlnaCA8LSBzZXRbd2hpY2gocmVzaWQgPiBxdWFudGlsZShyZXNpZCwgMS8yKSksXQ0KICAgICAgbG93IDwtIHNldFt3aGljaChyZXNpZCA8IHF1YW50aWxlKHJlc2lkLDEvMikpLF0NCiAgICAgIGhpZ2ggPC0gaGlnaFssLTFdDQogICAgICBsb3cgPC0gbG93WywtMV0NCiAgICAgIA0KICAgICAgDQogICAgICBpZihhYnMoY29yKGhpZ2hbLDFdLGhpZ2hbLDJdKSkgPCBhKXsNCiAgICAgICAgaCA8LSAwDQogICAgICB9ZWxzZXsNCiAgICAgICAgaCA8LSBjb3IoaGlnaFssMV0saGlnaFssMl0pDQogICAgICB9DQogICAgICANCiAgICAgIGlmKGFicyhjb3IobG93WywxXSxsb3dbLDJdKSkgPCBhKXsNCiAgICAgICAgTCA8LSAwDQogICAgICB9ZWxzZXsNCiAgICAgICAgTCA8LSBjb3IobG93WywxXSxsb3dbLDJdKQ0KICAgICAgfQ0KICAgICAgDQogICAgICBkUENDTltpLGpdIDwtIGgtTA0KICAgICAgDQogICAgICBzaWduUFtpLGpdIDwtIDAuNSphYnMoKHNpZ24oaCktc2lnbihMKSkpDQogICAgICANCiAgICAgIA0KICAgIH0NCiAgfQ0KICANCiAgZGlhZyhkUENDTikgPC0gMA0KICANCiAgc2lnblBbc2lnblAgIT0gMCBdIDwtIDENCiAgc2lnbmRQQ0NOIDwtIGFicyhzaWduUCpkUENDTikNCiAgDQogIA0KICANCiAgDQogICNDYWxjdWxhdGUgQVVDUyBmb3IgZWFjaCByZXBsaWNhdGlvbg0KICANCiAgZENDTmF1YzAyW2xdIDwtIHJvYyhST0NtYXRyaXhbdXBwZXIudHJpKFJPQ21hdHJpeCldLGRDQ05bdXBwZXIudHJpKGRDQ04pXSwgcGFydGlhbC5hdWMgPSBjKDAuOCwxLjApLCBwYXJ0aWFsLmF1Yy5jb3JyZWN0ID0gVFJVRSkkYXVjDQogIGRQQ0NOYXVjMDJbbF08LSByb2MoUk9DbWF0cml4W3VwcGVyLnRyaShST0NtYXRyaXgpXSxkUENDTlt1cHBlci50cmkoZFBDQ04pXSwgcGFydGlhbC5hdWMgPSBjKDAuOCwxLjApLCBwYXJ0aWFsLmF1Yy5jb3JyZWN0ID0gVFJVRSkkYXVjDQogIHNpZ25kQ0NOYXVjMDJbbF08LSByb2MoUk9DbWF0cml4W3VwcGVyLnRyaShST0NtYXRyaXgpXSxzaWduZENDTlt1cHBlci50cmkoc2lnbmRDQ04pXSwgcGFydGlhbC5hdWMgPSBjKDAuOCwxLjApLCBwYXJ0aWFsLmF1Yy5jb3JyZWN0ID0gVFJVRSkkYXVjDQogIHNpZ25kUENDTmF1YzAyW2xdPC0gcm9jKFJPQ21hdHJpeFt1cHBlci50cmkoUk9DbWF0cml4KV0sc2lnbmRQQ0NOW3VwcGVyLnRyaShzaWduZFBDQ04pXSwgcGFydGlhbC5hdWMgPSBjKDAuOCwxLjApLCBwYXJ0aWFsLmF1Yy5jb3JyZWN0ID0gVFJVRSkkYXVjDQogIGV4aGF1c3RpdmVhdWMwMltsXSA8LSByb2MoUk9DdmVjLGFicyhjdkNvZWZmaWNpZW50c1ssbF0pLHBhcnRpYWwuYXVjID0gYygwLjgsMS4wKSwgcGFydGlhbC5hdWMuY29ycmVjdCA9IFRSVUUpJGF1Yw0KICANCiAgZENDTmF1Y1tsXSA8LSByb2MoUk9DbWF0cml4W3VwcGVyLnRyaShST0NtYXRyaXgpXSxkQ0NOW3VwcGVyLnRyaShkQ0NOKV0pJGF1Yw0KICBkUENDTmF1Y1tsXTwtIHJvYyhST0NtYXRyaXhbdXBwZXIudHJpKFJPQ21hdHJpeCldLGRQQ0NOW3VwcGVyLnRyaShkUENDTildKSRhdWMNCiAgc2lnbmRDQ05hdWNbbF08LSByb2MoUk9DbWF0cml4W3VwcGVyLnRyaShST0NtYXRyaXgpXSxzaWduZENDTlt1cHBlci50cmkoc2lnbmRDQ04pXSkkYXVjDQogIHNpZ25kUENDTmF1Y1tsXTwtIHJvYyhST0NtYXRyaXhbdXBwZXIudHJpKFJPQ21hdHJpeCldLHNpZ25kUENDTlt1cHBlci50cmkoc2lnbmRQQ0NOKV0pJGF1Yw0KICBleGhhdXN0aXZlYXVjW2xdIDwtIHJvYyhST0N2ZWMsIGFicyhjdkNvZWZmaWNpZW50c1ssbF0pKSRhdWMNCiAgDQogIA0KICBkR0dNYXVjW2xdIDwtICByb2MoUk9DbWF0cml4W3VwcGVyLnRyaShST0NtYXRyaXgpXSxHR01bdXBwZXIudHJpKGFzLm1hdHJpeChHR00pKV0pJGF1Yw0KICBkR0dNYXVjMDJbbF0gPC0gIHJvYyhST0NtYXRyaXhbdXBwZXIudHJpKFJPQ21hdHJpeCldLEdHTVt1cHBlci50cmkoYXMubWF0cml4KEdHTSkpXSwgcGFydGlhbC5hdWMgPSBjKDAuOCwxLjApLCBwYXJ0aWFsLmF1Yy5jb3JyZWN0ID0gVFJVRSkkYXVjDQogIA0KICANCiAgDQogICMjIyMjIyMjIyMjIyMjIyMjIyMjIyMjIyMjIyMjIyMjIyMjIyMjIyMNCiAgDQogIA0KICBsb3cuY2kuZENDTmF1YzAyW2xdIDwtIGNpLmF1Yyhyb2MoUk9DbWF0cml4W3VwcGVyLnRyaShST0NtYXRyaXgpXSxkQ0NOW3VwcGVyLnRyaShkQ0NOKV0sIHBhcnRpYWwuYXVjID0gYygwLjgsMS4wKSwgcGFydGlhbC5hdWMuY29ycmVjdCA9IFRSVUUpJGF1YylbMV0NCiAgbG93LmNpLmRQQ0NOYXVjMDJbbF08LSBjaS5hdWMocm9jKFJPQ21hdHJpeFt1cHBlci50cmkoUk9DbWF0cml4KV0sZFBDQ05bdXBwZXIudHJpKGRQQ0NOKV0sIHBhcnRpYWwuYXVjID0gYygwLjgsMS4wKSwgcGFydGlhbC5hdWMuY29ycmVjdCA9IFRSVUUpJGF1YylbMV0NCiAgbG93LmNpLnNpZ25kQ0NOYXVjMDJbbF08LSBjaS5hdWMocm9jKFJPQ21hdHJpeFt1cHBlci50cmkoUk9DbWF0cml4KV0sc2lnbmRDQ05bdXBwZXIudHJpKHNpZ25kQ0NOKV0sIHBhcnRpYWwuYXVjID0gYygwLjgsMS4wKSwgcGFydGlhbC5hdWMuY29ycmVjdCA9IFRSVUUpJGF1YylbMV0NCiAgbG93LmNpLnNpZ25kUENDTmF1YzAyW2xdPC0gY2kuYXVjKHJvYyhST0NtYXRyaXhbdXBwZXIudHJpKFJPQ21hdHJpeCldLHNpZ25kUENDTlt1cHBlci50cmkoc2lnbmRQQ0NOKV0sIHBhcnRpYWwuYXVjID0gYygwLjgsMS4wKSwgcGFydGlhbC5hdWMuY29ycmVjdCA9IFRSVUUpJGF1YylbMV0NCiAgbG93LmNpLmV4aGF1c3RpdmVhdWMwMltsXSA8LSBjaS5hdWMocm9jKFJPQ3ZlYyxhYnMoY3ZDb2VmZmljaWVudHNbLGxdKSxwYXJ0aWFsLmF1YyA9IGMoMC44LDEuMCksIHBhcnRpYWwuYXVjLmNvcnJlY3QgPSBUUlVFKSRhdWMpWzFdDQogIA0KICBsb3cuY2kuZENDTmF1Y1tsXSA8LSBjaS5hdWMocm9jKFJPQ21hdHJpeFt1cHBlci50cmkoUk9DbWF0cml4KV0sZENDTlt1cHBlci50cmkoZENDTildKSRhdWMpWzFdDQogIGxvdy5jaS5kUENDTmF1Y1tsXTwtIGNpLmF1Yyhyb2MoUk9DbWF0cml4W3VwcGVyLnRyaShST0NtYXRyaXgpXSxkUENDTlt1cHBlci50cmkoZFBDQ04pXSkkYXVjKVsxXQ0KICBsb3cuY2kuc2lnbmRDQ05hdWNbbF08LSBjaS5hdWMocm9jKFJPQ21hdHJpeFt1cHBlci50cmkoUk9DbWF0cml4KV0sc2lnbmRDQ05bdXBwZXIudHJpKHNpZ25kQ0NOKV0pJGF1YylbMV0NCiAgbG93LmNpLnNpZ25kUENDTmF1Y1tsXTwtIGNpLmF1Yyhyb2MoUk9DbWF0cml4W3VwcGVyLnRyaShST0NtYXRyaXgpXSxzaWduZFBDQ05bdXBwZXIudHJpKHNpZ25kUENDTildKSRhdWMpWzFdDQogIGxvdy5jaS5leGhhdXN0aXZlYXVjW2xdIDwtIGNpLmF1Yyhyb2MoUk9DdmVjLCBhYnMoY3ZDb2VmZmljaWVudHNbLGxdKSkkYXVjKVsxXQ0KICANCiAgDQogIGxvdy5jaS5kR0dNYXVjW2xdIDwtICBjaS5hdWMocm9jKFJPQ21hdHJpeFt1cHBlci50cmkoUk9DbWF0cml4KV0sR0dNW3VwcGVyLnRyaShhcy5tYXRyaXgoR0dNKSldKSRhdWMpWzFdDQogIGxvdy5jaS5kR0dNYXVjMDJbbF0gPC0gIGNpLmF1Yyhyb2MoUk9DbWF0cml4W3VwcGVyLnRyaShST0NtYXRyaXgpXSxHR01bdXBwZXIudHJpKGFzLm1hdHJpeChHR00pKV0sIHBhcnRpYWwuYXVjID0gYygwLjgsMS4wKSwgcGFydGlhbC5hdWMuY29ycmVjdCA9IFRSVUUpJGF1YylbMV0NCiAgDQogIA0KICAjIyMjIyMjIyMjIyMjIyMjIyMjIyMjIyMjIyMjIyMjIyMjIyMjIyMjIyMjIyMjIyMjIyMjIyMjDQogIA0KICBoaWdoLmNpLmRDQ05hdWMwMltsXSA8LSBjaS5hdWMocm9jKFJPQ21hdHJpeFt1cHBlci50cmkoUk9DbWF0cml4KV0sZENDTlt1cHBlci50cmkoZENDTildLCBwYXJ0aWFsLmF1YyA9IGMoMC44LDEuMCksIHBhcnRpYWwuYXVjLmNvcnJlY3QgPSBUUlVFKSRhdWMpWzNdDQogIGhpZ2guY2kuZFBDQ05hdWMwMltsXTwtIGNpLmF1Yyhyb2MoUk9DbWF0cml4W3VwcGVyLnRyaShST0NtYXRyaXgpXSxkUENDTlt1cHBlci50cmkoZFBDQ04pXSwgcGFydGlhbC5hdWMgPSBjKDAuOCwxLjApLCBwYXJ0aWFsLmF1Yy5jb3JyZWN0ID0gVFJVRSkkYXVjKVszXQ0KICBoaWdoLmNpLnNpZ25kQ0NOYXVjMDJbbF08LSBjaS5hdWMocm9jKFJPQ21hdHJpeFt1cHBlci50cmkoUk9DbWF0cml4KV0sc2lnbmRDQ05bdXBwZXIudHJpKHNpZ25kQ0NOKV0sIHBhcnRpYWwuYXVjID0gYygwLjgsMS4wKSwgcGFydGlhbC5hdWMuY29ycmVjdCA9IFRSVUUpJGF1YylbM10NCiAgaGlnaC5jaS5zaWduZFBDQ05hdWMwMltsXTwtIGNpLmF1Yyhyb2MoUk9DbWF0cml4W3VwcGVyLnRyaShST0NtYXRyaXgpXSxzaWduZFBDQ05bdXBwZXIudHJpKHNpZ25kUENDTildLCBwYXJ0aWFsLmF1YyA9IGMoMC44LDEuMCksIHBhcnRpYWwuYXVjLmNvcnJlY3QgPSBUUlVFKSRhdWMpWzNdDQogIGhpZ2guY2kuZXhoYXVzdGl2ZWF1YzAyW2xdIDwtIGNpLmF1Yyhyb2MoUk9DdmVjLGFicyhjdkNvZWZmaWNpZW50c1ssbF0pLHBhcnRpYWwuYXVjID0gYygwLjgsMS4wKSwgcGFydGlhbC5hdWMuY29ycmVjdCA9IFRSVUUpJGF1YylbM10NCiAgDQogIGhpZ2guY2kuZENDTmF1Y1tsXSA8LSBjaS5hdWMocm9jKFJPQ21hdHJpeFt1cHBlci50cmkoUk9DbWF0cml4KV0sZENDTlt1cHBlci50cmkoZENDTildKSRhdWMpWzNdDQogIGhpZ2guY2kuZFBDQ05hdWNbbF08LSBjaS5hdWMocm9jKFJPQ21hdHJpeFt1cHBlci50cmkoUk9DbWF0cml4KV0sZFBDQ05bdXBwZXIudHJpKGRQQ0NOKV0pJGF1YylbM10NCiAgaGlnaC5jaS5zaWduZENDTmF1Y1tsXTwtIGNpLmF1Yyhyb2MoUk9DbWF0cml4W3VwcGVyLnRyaShST0NtYXRyaXgpXSxzaWduZENDTlt1cHBlci50cmkoc2lnbmRDQ04pXSkkYXVjKVszXQ0KICBoaWdoLmNpLnNpZ25kUENDTmF1Y1tsXTwtIGNpLmF1Yyhyb2MoUk9DbWF0cml4W3VwcGVyLnRyaShST0NtYXRyaXgpXSxzaWduZFBDQ05bdXBwZXIudHJpKHNpZ25kUENDTildKSRhdWMpWzNdDQogIGhpZ2guY2kuZXhoYXVzdGl2ZWF1Y1tsXSA8LSBjaS5hdWMocm9jKFJPQ3ZlYywgYWJzKGN2Q29lZmZpY2llbnRzWyxsXSkpJGF1YylbM10NCiAgDQogIA0KICBoaWdoLmNpLmRHR01hdWNbbF0gPC0gIGNpLmF1Yyhyb2MoUk9DbWF0cml4W3VwcGVyLnRyaShST0NtYXRyaXgpXSxHR01bdXBwZXIudHJpKGFzLm1hdHJpeChHR00pKV0pJGF1YylbM10NCiAgaGlnaC5jaS5kR0dNYXVjMDJbbF0gPC0gIGNpLmF1Yyhyb2MoUk9DbWF0cml4W3VwcGVyLnRyaShST0NtYXRyaXgpXSxHR01bdXBwZXIudHJpKGFzLm1hdHJpeChHR00pKV0sIHBhcnRpYWwuYXVjID0gYygwLjgsMS4wKSwgcGFydGlhbC5hdWMuY29ycmVjdCA9IFRSVUUpJGF1YylbM10NCiAgDQogIH0NCg0KYGBgDQoNCiNBVkVSQUdFIFRIRSBSRVNVTFRTIE9WRVIgUkVQTElDQVRJT05TDQpgYGB7cn0NCmMobWVhbihkQ0NOYXVjMDIpLCBtZWFuKGxvdy5jaS5kQ0NOYXVjMDIpLG1lYW4oaGlnaC5jaS5kQ0NOYXVjMDIpKQ0KYyhtZWFuKHNpZ25kQ0NOYXVjMDIsbmEucm09VFJVRSksIG1lYW4obG93LmNpLnNpZ25kQ0NOYXVjMDIpLG1lYW4oaGlnaC5jaS5zaWduZENDTmF1YzAyKSkNCmMobWVhbihzaWduZFBDQ05hdWMwMiksIG1lYW4obG93LmNpLnNpZ25kUENDTmF1YzAyKSxtZWFuKGhpZ2guY2kuc2lnbmRQQ0NOYXVjMDIpKQ0KYyhtZWFuKGV4aGF1c3RpdmVhdWMwMixuYS5ybT1UUlVFKSwgbWVhbihsb3cuY2kuZXhoYXVzdGl2ZWF1YzAyKSxtZWFuKGhpZ2guY2kuZXhoYXVzdGl2ZWF1YzAyKSkNCmMobWVhbihkR0dNYXVjMDIpLCBtZWFuKGxvdy5jaS5kR0dNYXVjMDIpLG1lYW4oaGlnaC5jaS5kR0dNYXVjMDIpKQ0KDQoNCg0KYyhtZWFuKGRDQ05hdWMpLCBtZWFuKGxvdy5jaS5kQ0NOYXVjKSxtZWFuKGhpZ2guY2kuZENDTmF1YykpDQpjKG1lYW4oc2lnbmRDQ05hdWMpLCBtZWFuKGxvdy5jaS5zaWduZENDTmF1YyksbWVhbihoaWdoLmNpLnNpZ25kQ0NOYXVjKSkNCmMobWVhbihzaWduZFBDQ05hdWMpLCBtZWFuKGxvdy5jaS5zaWduZFBDQ05hdWMpLG1lYW4oaGlnaC5jaS5zaWduZFBDQ05hdWMpKQ0KYyhtZWFuKGV4aGF1c3RpdmVhdWMpLCBtZWFuKGxvdy5jaS5leGhhdXN0aXZlYXVjKSxtZWFuKGhpZ2guY2kuZXhoYXVzdGl2ZWF1YykpDQpjKG1lYW4oZEdHTWF1YyksIG1lYW4obG93LmNpLmRHR01hdWMpLG1lYW4oaGlnaC5jaS5kR0dNYXVjKSkNCmBgYA0KDQo=
