# Supplementary material for: Model guided trait-specific co-expression network estimation as a new perspective for identifying molecular interactions and pathways
Source: PLoS Comput Biol. 2021 May 3;17(5):e1008960. doi: 10.1371/journal.pcbi.1008960 (PMC8118548; doi:10.1371/journal.pcbi.1008960)
Supplement: S2 Appendix — See also a GitHub repository https://github.com/JAJKontio/model_diffnet.git. (ZIP) [file pcbi.1008960.s002.zip › Preview_documents/SIMULATED_EXAMPLES_MODEL_B.nb.html]

SIMULATED EXAMPLES: MODEL B


Code 

- Show All Code
- Hide All Code
- Download Rmd

# SIMULATED EXAMPLES: MODEL B

Go to a website (https://www.synapse.org/#!Synapse:syn2455683/wiki/64007) and download a normalized protein expression DREAM9-challenge dataset (trainingData-release.csv) (Noren et al.2016 - https://doi.org/10.1371/journal.pcbi.1004890) which is available upon registration (http://dreamchallenges.org/). These data were provided by Dr. Steven Kornblau from the University of Texas MD Anderson Cancer Center and were obtained through Synapse syn2455683 as a part of the acute myeloid leukemia (AML) DREAM-challenge.

#STEP 0: PREPARING THE DATASET AND INSTALLING THE REQUIRED R-PACKAGES Install the required packages and prepare the AML dataset from the downloaded CSV-file “trainingData-release.csv” (requires registration) - https://www.synapse.org/#!Synapse:syn2455683/wiki/64007.


```
#DOWNLOAD REQUIRED R-PACKAGES
library("glmnet") #https://CRAN.R-project.org/package=glmnet
library("matrixStats") #https://CRAN.R-project.org/package=matrixStats
library("qgraph") #https://CRAN.R-project.org/package=qgraph 
library("netdiffuseR") #https://CRAN.R-project.org/package=netdiffuseR
library("stringr") #https://CRAN.R-project.org/package=stringr 

#Replace "~/trainingData-release.csv" with a user-specific path to the downloaded CSV-file.
data <-read.csv("~/trainingData-release.csv", header = TRUE)

#Separate the expression data (renamed as "rdata") from clinical covariates.
rdata <- data[,c(42:272)]
```


#INITIALIZE


```
#Number of replicates
k = 10
data <- rdata
#Empty list and vectors for replicates
dCCNlist <- rep(list(matrix(NA, ncol(rdata), ncol(rdata)), k))
dPCCNlist <- rep(list(matrix(NA, ncol(rdata), ncol(rdata)), k))
GGMlist <- rep(list(matrix(NA, ncol(rdata), ncol(rdata)), k))
ymat <- matrix(0,191,k)
her1 <- rep(0,k)
rdatareps <- rep(list(matrix(NA, ncol(rdata), ncol(rdata))), k)

#Empty vectors for AUCS and their confidence intervals calculated over replicates

dCCNauc <- rep(0,k)
dPCCNauc<- rep(0,k)
signdCCNauc<- rep(0,k)
signdPCCNauc<- rep(0,k)
exhaustiveauc <- rep(0,k)
dGGMauc <- rep(0,k)

dCCNauc02 <- rep(0,k)
dPCCNauc02<- rep(0,k)
signdCCNauc02<- rep(0,k)
signdPCCNauc02<- rep(0,k)
exhaustiveauc02 <- rep(0,k)
dGGMauc02 <- rep(0,k)

################################################
low.ci.dCCNauc <- rep(0,k)
low.ci.dPCCNauc<- rep(0,k)
low.ci.signdCCNauc<- rep(0,k)
low.ci.signdPCCNauc<- rep(0,k)
low.ci.exhaustiveauc <- rep(0,k)
low.ci.dGGMauc <- rep(0,k)

low.ci.dCCNauc02 <- rep(0,k)
low.ci.dPCCNauc02<- rep(0,k)
low.ci.signdCCNauc02<- rep(0,k)
low.ci.signdPCCNauc02<- rep(0,k)
low.ci.exhaustiveauc02 <- rep(0,k)
low.ci.dGGMauc02 <- rep(0,k)
################################################

high.ci.dCCNauc <- rep(0,k)
high.ci.dPCCNauc<- rep(0,k)
high.ci.signdCCNauc<- rep(0,k)
high.ci.signdPCCNauc<- rep(0,k)
high.ci.exhaustiveauc <- rep(0,k)
high.ci.dGGMauc <- rep(0,k)

high.ci.dCCNauc02 <- rep(0,k)
high.ci.dPCCNauc02<- rep(0,k)
high.ci.signdCCNauc02<- rep(0,k)
high.ci.signdPCCNauc02<- rep(0,k)
high.ci.exhaustiveauc02 <- rep(0,k)
high.ci.dGGMauc02 <- rep(0,k)

####################################################


#Indicate the true positives


ROCmatrix <- matrix(0,ncol(rdata),ncol(rdata))

ROCmatrix[75,150] <- 1
ROCmatrix[100,200] <- 1
ROCmatrix[125,215] <- 1
ROCmatrix[25,52] <- 1

ROCmatrix[33,66] <- 1
ROCmatrix[88,144] <- 1

ROCmatrix[2,170] <- 1
ROCmatrix[50,115] <- 1
ROCmatrix[44,99] <- 1

ROCmatrix[12,180] <- 1
ROCmatrix[60,125] <- 1
ROCmatrix[22,190] <- 1
ROCmatrix[22,211] <- 1


ROCmatrix[70,135] <- 1
ROCmatrix[32,221] <- 1
ROCmatrix[12,183] <- 1
ROCmatrix[54,109] <- 1

#True positive for the exhaustive search

observations <-read.csv("~/trainingData-release.csv", header = FALSE)
  observations <- observations[-1,c(42:272)]
  nams <- apply( combn(colnames(observations),2), 2, function(z) paste(z, collapse = '*'))
  cols <- combn(ncol(rdata), 2)
  three <- apply(cols, 2, function(z) rowProds(as.matrix(rdata)[,z]))
  colnames(three) <- nams
  
  
ROCvec <- rep(0,dim(three)[2])
cvCoefficients <- matrix(0,dim(three)[2],k)
ROCvec[which(colnames(three) == "V116*V191")] <- 1
ROCvec[which(colnames(three) == "V141*V241")] <- 1
ROCvec[which(colnames(three) == "V166*V256")] <- 1
ROCvec[which(colnames(three) == "V66*V93")] <- 1


ROCvec[which(colnames(three) == "V74*V107")] <- 1
ROCvec[which(colnames(three) == "V129*V185")] <- 1

ROCvec[which(colnames(three) == "V43*V211")] <- 1
ROCvec[which(colnames(three) == "V91*V156")] <- 1
ROCvec[which(colnames(three) == "V85*V140")] <- 1

ROCvec[which(colnames(three) == "V53*V221")] <- 1
ROCvec[which(colnames(three) == "V101*V166")] <- 1
ROCvec[which(colnames(three) == "V63*V231")] <- 1
ROCvec[which(colnames(three) == "V63*V252")] <- 1


ROCvec[which(colnames(three) == "V111*V176")] <- 1
ROCvec[which(colnames(three) == "V73*V262")] <- 1
ROCvec[which(colnames(three) == "V53*V224")] <- 1
ROCvec[which(colnames(three) == "V95*V150")] <- 1
```


#SIMULATE THE MODELS AND PERFORM ALL ANALYSES


```
#####################SIMULATED MODEL#####################


#TWO RELU TERMS

z1 <- rdata[,12]*rdata[,183]
z2 <- rdata[,109]*rdata[,54]

z1[which(z1 < quantile(z1, 0.75))] <-0
z2[which(z2 < quantile(z2, 0.75))] <-0


ymat <- matrix(0,191,k)
for(l in 1:k){
  
  data <-read.csv("rr.csv", header = TRUE)
  rdata <- data[,c(42:272)]
  
  rdata[,125] <- rdata[,215]  + rnorm(191,0,0.25)
  rdata[,75] <- rdata[,150] + rnorm(191,0,0.25)
  
  
  ymat[,l] <- rdata[,75]*rdata[,150]+rdata[,100]*rdata[,200] +rdata[,125]*rdata[,215] +rdata[,25]*rdata[,52]+rdata[,33]*rdata[,66]+rdata[,88]*rdata[,144]+
    2*(z1+z2)+rnorm(191,0,1.75)
  y1 <- ymat[,l]
  her1[l] <- (var(y1)-1.75^2)/var(y1)
  
  rdata[which(y1 < quantile(y1, 0.80)),2] <- -rdata[which(y1 < quantile(y1, 0.80)),170] + rnorm(length(which(y1 < quantile(y1, 0.80))),0,0.25)
  rdata[which(y1 < quantile(y1, 0.80)),50] <- -rdata[which(y1 < quantile(y1, 0.80)),115] + rnorm(length(which(y1 < quantile(y1, 0.80))),0,0.25)
  rdata[which(y1 > quantile(y1, 0.80)),99] <- -rdata[which(y1 > quantile(y1, 0.80)),44] + rnorm(length(which(y1 > quantile(y1, 0.80))),0,0.25)
  
  rdata[which(y1 < quantile(y1, 0.80)),12] <- rdata[which(y1 < quantile(y1, 0.80)),180] + rnorm(length(which(y1 < quantile(y1, 0.80))),0,0.25)
  rdata[which(y1 < quantile(y1, 0.80)),60] <- rdata[which(y1 < quantile(y1, 0.80)),125] + rnorm(length(which(y1 < quantile(y1, 0.80))),0,0.25)
  rdata[which(y1 < quantile(y1, 0.80)),22] <- rdata[which(y1 < quantile(y1, 0.80)),211] + rnorm(length(which(y1 < quantile(y1, 0.80))),0,0.25)
  
  rdata[which(y1 < quantile(y1, 0.80)),22] <- rdata[which(y1 < quantile(y1, 0.80)),190] + rnorm(length(which(y1 < quantile(y1, 0.80))),0,0.25)
  rdata[which(y1 < quantile(y1, 0.80)),70] <- rdata[which(y1 < quantile(y1, 0.80)),135] + rnorm(length(which(y1 < quantile(y1, 0.80))),0,0.25)
  rdata[which(y1 < quantile(y1, 0.80)),32] <- rdata[which(y1 < quantile(y1, 0.80)),221] + rnorm(length(which(y1 < quantile(y1, 0.80))),0,0.25)
  
  ymat[,l] <- ymat[,l] + 2*(rdata[,10] + rdata[,30] +rdata[,50] + rdata[,70] + rdata[,90] + rdata[,100])
  y1 <- ymat[,l]
  
  
  data <- rdata
  rdatareps[[l]] <- rdata 
  
  
  
  
 #####EXHAUSTIVE SEARCH########################
  
  #Enumerate all possible pairwise interactions
  
  observations <- read.csv("~/trainingData-release.csv", header = TRUE)
  observations <- observations[-1,c(42:272)]
  nams <- apply( combn(colnames(observations),2), 2, function(z) paste(z, collapse = '*'))
  cols <- combn(ncol(rdata), 2)
  three <- apply(cols, 2, function(z) rowProds(as.matrix(rdata)[,z]))
  colnames(three) <- nams
  
  cv.fit=cv.glmnet(three, y1, alpha = 0, maxit = 10000)
  fit=glmnet(three,y1, alpha = 0, maxit = 10000)
  cvCoefficients[,l] <- coef(fit, s = cv.fit$lambda.min)[-1]
  
  
  
  
  
  
  
  ############differential GGM#########
  high <- rdata[which(y1 > quantile(y1, 0.5)),]
  low <- rdata[which(y1 < quantile(y1, 0.5)),]
  fgl.results = JGL(Y=list(low, high),penalty="fused",lambda1=.1,lambda2=.1,return.whole.theta=TRUE)
  GGM <-abs(as.matrix(fgl.results$theta[[1]])-as.matrix(fgl.results$theta[[2]]))
  
  
  ########################
  ###differential DCCN####
  dCCN <- abs(cor(high) - cor(low))
  signdCCN <- (abs(sign(cor(high))-sign(cor(low))))*dCCN
  
  ########################
  
  
  
  
  
  
  ############Sign-adjusted dPCCN#################
  
  #Residual step
  rdata <- as.matrix(rdata)
  cv.fit <- cv.glmnet(rdata, scale(y1), alpha = 1.0, maxit = 10000)
  fit=glmnet(rdata, scale(y1), alpha = 1.0, maxit = 10000)
  Coefficients <- coef(fit, s = cv.fit$lambda.min)
  v <- rdata %*% Coefficients[-1]
  resid <- scale(y1)-v
  
  
  #Estimate dPCCN structures
  a = 0.1
  dPCCN <- matrix(0,ncol(data),ncol(data))
  signP <- matrix(0,ncol(data),ncol(data))
  
  
  for(i in 1:ncol(data)){
    for(j in 1:ncol(data)){
      
      res <- (lm(rdata[,j]~rdata[,i])$residuals)
      set <- cbind(resid,res,rdata[,i])
      high <- set[which(resid > quantile(resid, 1/2)),]
      low <- set[which(resid < quantile(resid,1/2)),]
      high <- high[,-1]
      low <- low[,-1]
      
      
      if(abs(cor(high[,1],high[,2])) < a){
        h <- 0
      }else{
        h <- cor(high[,1],high[,2])
      }
      
      if(abs(cor(low[,1],low[,2])) < a){
        L <- 0
      }else{
        L <- cor(low[,1],low[,2])
      }
      
      dPCCN[i,j] <- h-L
      
      signP[i,j] <- 0.5*abs((sign(h)-sign(L)))
      
      
    }
  }
  
  diag(dPCCN) <- 0
  
  signP[signP != 0 ] <- 1
  signdPCCN <- abs(signP*dPCCN)
  
  

  
  #Calculate AUCS for each replication
  
  dCCNauc02[l] <- roc(ROCmatrix[upper.tri(ROCmatrix)],dCCN[upper.tri(dCCN)], partial.auc = c(0.8,1.0), partial.auc.correct = TRUE)$auc
  dPCCNauc02[l]<- roc(ROCmatrix[upper.tri(ROCmatrix)],dPCCN[upper.tri(dPCCN)], partial.auc = c(0.8,1.0), partial.auc.correct = TRUE)$auc
  signdCCNauc02[l]<- roc(ROCmatrix[upper.tri(ROCmatrix)],signdCCN[upper.tri(signdCCN)], partial.auc = c(0.8,1.0), partial.auc.correct = TRUE)$auc
  signdPCCNauc02[l]<- roc(ROCmatrix[upper.tri(ROCmatrix)],signdPCCN[upper.tri(signdPCCN)], partial.auc = c(0.8,1.0), partial.auc.correct = TRUE)$auc
  exhaustiveauc02[l] <- roc(ROCvec,abs(cvCoefficients[,l]),partial.auc = c(0.8,1.0), partial.auc.correct = TRUE)$auc
  
  dCCNauc[l] <- roc(ROCmatrix[upper.tri(ROCmatrix)],dCCN[upper.tri(dCCN)])$auc
  dPCCNauc[l]<- roc(ROCmatrix[upper.tri(ROCmatrix)],dPCCN[upper.tri(dPCCN)])$auc
  signdCCNauc[l]<- roc(ROCmatrix[upper.tri(ROCmatrix)],signdCCN[upper.tri(signdCCN)])$auc
  signdPCCNauc[l]<- roc(ROCmatrix[upper.tri(ROCmatrix)],signdPCCN[upper.tri(signdPCCN)])$auc
  exhaustiveauc[l] <- roc(ROCvec, abs(cvCoefficients[,l]))$auc
  
  
  dGGMauc[l] <-  roc(ROCmatrix[upper.tri(ROCmatrix)],GGM[upper.tri(as.matrix(GGM))])$auc
  dGGMauc02[l] <-  roc(ROCmatrix[upper.tri(ROCmatrix)],GGM[upper.tri(as.matrix(GGM))], partial.auc = c(0.8,1.0), partial.auc.correct = TRUE)$auc
  
  
  
########################################
  
  
  low.ci.dCCNauc02[l] <- ci.auc(roc(ROCmatrix[upper.tri(ROCmatrix)],dCCN[upper.tri(dCCN)], partial.auc = c(0.8,1.0), partial.auc.correct = TRUE)$auc)[1]
  low.ci.dPCCNauc02[l]<- ci.auc(roc(ROCmatrix[upper.tri(ROCmatrix)],dPCCN[upper.tri(dPCCN)], partial.auc = c(0.8,1.0), partial.auc.correct = TRUE)$auc)[1]
  low.ci.signdCCNauc02[l]<- ci.auc(roc(ROCmatrix[upper.tri(ROCmatrix)],signdCCN[upper.tri(signdCCN)], partial.auc = c(0.8,1.0), partial.auc.correct = TRUE)$auc)[1]
  low.ci.signdPCCNauc02[l]<- ci.auc(roc(ROCmatrix[upper.tri(ROCmatrix)],signdPCCN[upper.tri(signdPCCN)], partial.auc = c(0.8,1.0), partial.auc.correct = TRUE)$auc)[1]
  low.ci.exhaustiveauc02[l] <- ci.auc(roc(ROCvec,abs(cvCoefficients[,l]),partial.auc = c(0.8,1.0), partial.auc.correct = TRUE)$auc)[1]
  
  low.ci.dCCNauc[l] <- ci.auc(roc(ROCmatrix[upper.tri(ROCmatrix)],dCCN[upper.tri(dCCN)])$auc)[1]
  low.ci.dPCCNauc[l]<- ci.auc(roc(ROCmatrix[upper.tri(ROCmatrix)],dPCCN[upper.tri(dPCCN)])$auc)[1]
  low.ci.signdCCNauc[l]<- ci.auc(roc(ROCmatrix[upper.tri(ROCmatrix)],signdCCN[upper.tri(signdCCN)])$auc)[1]
  low.ci.signdPCCNauc[l]<- ci.auc(roc(ROCmatrix[upper.tri(ROCmatrix)],signdPCCN[upper.tri(signdPCCN)])$auc)[1]
  low.ci.exhaustiveauc[l] <- ci.auc(roc(ROCvec, abs(cvCoefficients[,l]))$auc)[1]
  
  
  low.ci.dGGMauc[l] <-  ci.auc(roc(ROCmatrix[upper.tri(ROCmatrix)],GGM[upper.tri(as.matrix(GGM))])$auc)[1]
  low.ci.dGGMauc02[l] <-  ci.auc(roc(ROCmatrix[upper.tri(ROCmatrix)],GGM[upper.tri(as.matrix(GGM))], partial.auc = c(0.8,1.0), partial.auc.correct = TRUE)$auc)[1]
  
  
#######################################################
  
  high.ci.dCCNauc02[l] <- ci.auc(roc(ROCmatrix[upper.tri(ROCmatrix)],dCCN[upper.tri(dCCN)], partial.auc = c(0.8,1.0), partial.auc.correct = TRUE)$auc)[3]
  high.ci.dPCCNauc02[l]<- ci.auc(roc(ROCmatrix[upper.tri(ROCmatrix)],dPCCN[upper.tri(dPCCN)], partial.auc = c(0.8,1.0), partial.auc.correct = TRUE)$auc)[3]
  high.ci.signdCCNauc02[l]<- ci.auc(roc(ROCmatrix[upper.tri(ROCmatrix)],signdCCN[upper.tri(signdCCN)], partial.auc = c(0.8,1.0), partial.auc.correct = TRUE)$auc)[3]
  high.ci.signdPCCNauc02[l]<- ci.auc(roc(ROCmatrix[upper.tri(ROCmatrix)],signdPCCN[upper.tri(signdPCCN)], partial.auc = c(0.8,1.0), partial.auc.correct = TRUE)$auc)[3]
  high.ci.exhaustiveauc02[l] <- ci.auc(roc(ROCvec,abs(cvCoefficients[,l]),partial.auc = c(0.8,1.0), partial.auc.correct = TRUE)$auc)[3]
  
  high.ci.dCCNauc[l] <- ci.auc(roc(ROCmatrix[upper.tri(ROCmatrix)],dCCN[upper.tri(dCCN)])$auc)[3]
  high.ci.dPCCNauc[l]<- ci.auc(roc(ROCmatrix[upper.tri(ROCmatrix)],dPCCN[upper.tri(dPCCN)])$auc)[3]
  high.ci.signdCCNauc[l]<- ci.auc(roc(ROCmatrix[upper.tri(ROCmatrix)],signdCCN[upper.tri(signdCCN)])$auc)[3]
  high.ci.signdPCCNauc[l]<- ci.auc(roc(ROCmatrix[upper.tri(ROCmatrix)],signdPCCN[upper.tri(signdPCCN)])$auc)[3]
  high.ci.exhaustiveauc[l] <- ci.auc(roc(ROCvec, abs(cvCoefficients[,l]))$auc)[3]
  
  
  high.ci.dGGMauc[l] <-  ci.auc(roc(ROCmatrix[upper.tri(ROCmatrix)],GGM[upper.tri(as.matrix(GGM))])$auc)[3]
  high.ci.dGGMauc02[l] <-  ci.auc(roc(ROCmatrix[upper.tri(ROCmatrix)],GGM[upper.tri(as.matrix(GGM))], partial.auc = c(0.8,1.0), partial.auc.correct = TRUE)$auc)[3]
  
  
}
```


#AVERAGE THE RESULTS OVER REPLICATIONS


```
c(mean(dCCNauc02), mean(low.ci.dCCNauc02),mean(high.ci.dCCNauc02))
c(mean(signdCCNauc02,na.rm=TRUE), mean(low.ci.signdCCNauc02),mean(high.ci.signdCCNauc02))
c(mean(signdPCCNauc02), mean(low.ci.signdPCCNauc02),mean(high.ci.signdPCCNauc02))
c(mean(exhaustiveauc02,na.rm=TRUE), mean(low.ci.exhaustiveauc02),mean(high.ci.exhaustiveauc02))
c(mean(dGGMauc02), mean(low.ci.dGGMauc02),mean(high.ci.dGGMauc02))

c(mean(dCCNauc), mean(low.ci.dCCNauc),mean(high.ci.dCCNauc))
c(mean(signdCCNauc), mean(low.ci.signdCCNauc),mean(high.ci.signdCCNauc))
c(mean(signdPCCNauc), mean(low.ci.signdPCCNauc),mean(high.ci.signdPCCNauc))
c(mean(exhaustiveauc), mean(low.ci.exhaustiveauc),mean(high.ci.exhaustiveauc))
c(mean(dGGMauc), mean(low.ci.dGGMauc),mean(high.ci.dGGMauc))
```


---


LS0tDQp0aXRsZTogIlNJTVVMQVRFRCBFWEFNUExFUzogTU9ERUwgQiINCm91dHB1dDogaHRtbF9ub3RlYm9vaw0KLS0tDQoNCg0KR28gdG8gYSB3ZWJzaXRlIChodHRwczovL3d3dy5zeW5hcHNlLm9yZy8jIVN5bmFwc2U6c3luMjQ1NTY4My93aWtpLzY0MDA3KSBhbmQgZG93bmxvYWQgYSBub3JtYWxpemVkIHByb3RlaW4gZXhwcmVzc2lvbiBEUkVBTTktY2hhbGxlbmdlIGRhdGFzZXQgKHRyYWluaW5nRGF0YS1yZWxlYXNlLmNzdikgKE5vcmVuIGV0IGFsLjIwMTYgLSBodHRwczovL2RvaS5vcmcvMTAuMTM3MS9qb3VybmFsLnBjYmkuMTAwNDg5MCkgd2hpY2ggaXMgYXZhaWxhYmxlIHVwb24gcmVnaXN0cmF0aW9uIChodHRwOi8vZHJlYW1jaGFsbGVuZ2VzLm9yZy8pLiBUaGVzZSBkYXRhIHdlcmUgcHJvdmlkZWQgYnkgRHIuIFN0ZXZlbiBLb3JuYmxhdSBmcm9tIHRoZSBVbml2ZXJzaXR5IG9mIFRleGFzIE1EIEFuZGVyc29uIENhbmNlciBDZW50ZXIgYW5kIHdlcmUgb2J0YWluZWQgdGhyb3VnaCBTeW5hcHNlIHN5bjI0NTU2ODMgYXMgYSBwYXJ0IG9mIHRoZSBhY3V0ZSBteWVsb2lkIGxldWtlbWlhIChBTUwpIERSRUFNLWNoYWxsZW5nZS4gDQoNCiNTVEVQIDA6IFBSRVBBUklORyBUSEUgREFUQVNFVCBBTkQgSU5TVEFMTElORyBUSEUgUkVRVUlSRUQgUi1QQUNLQUdFUw0KSW5zdGFsbCB0aGUgcmVxdWlyZWQgcGFja2FnZXMgYW5kIHByZXBhcmUgdGhlIEFNTCBkYXRhc2V0IGZyb20gdGhlIGRvd25sb2FkZWQgQ1NWLWZpbGUgInRyYWluaW5nRGF0YS1yZWxlYXNlLmNzdiIgKHJlcXVpcmVzIHJlZ2lzdHJhdGlvbikgLSBodHRwczovL3d3dy5zeW5hcHNlLm9yZy8jIVN5bmFwc2U6c3luMjQ1NTY4My93aWtpLzY0MDA3Lg0KDQpgYGB7cn0NCiNET1dOTE9BRCBSRVFVSVJFRCBSLVBBQ0tBR0VTDQpsaWJyYXJ5KCJnbG1uZXQiKSAjaHR0cHM6Ly9DUkFOLlItcHJvamVjdC5vcmcvcGFja2FnZT1nbG1uZXQNCmxpYnJhcnkoIm1hdHJpeFN0YXRzIikgI2h0dHBzOi8vQ1JBTi5SLXByb2plY3Qub3JnL3BhY2thZ2U9bWF0cml4U3RhdHMNCmxpYnJhcnkoInFncmFwaCIpICNodHRwczovL0NSQU4uUi1wcm9qZWN0Lm9yZy9wYWNrYWdlPXFncmFwaCANCmxpYnJhcnkoIm5ldGRpZmZ1c2VSIikgI2h0dHBzOi8vQ1JBTi5SLXByb2plY3Qub3JnL3BhY2thZ2U9bmV0ZGlmZnVzZVINCmxpYnJhcnkoInN0cmluZ3IiKSAjaHR0cHM6Ly9DUkFOLlItcHJvamVjdC5vcmcvcGFja2FnZT1zdHJpbmdyIA0KDQojUmVwbGFjZSAifi90cmFpbmluZ0RhdGEtcmVsZWFzZS5jc3YiIHdpdGggYSB1c2VyLXNwZWNpZmljIHBhdGggdG8gdGhlIGRvd25sb2FkZWQgQ1NWLWZpbGUuDQpkYXRhIDwtcmVhZC5jc3YoIn4vdHJhaW5pbmdEYXRhLXJlbGVhc2UuY3N2IiwgaGVhZGVyID0gVFJVRSkNCg0KI1NlcGFyYXRlIHRoZSBleHByZXNzaW9uIGRhdGEgKHJlbmFtZWQgYXMgInJkYXRhIikgZnJvbSBjbGluaWNhbCBjb3ZhcmlhdGVzLg0KcmRhdGEgPC0gZGF0YVssYyg0MjoyNzIpXQ0KDQpgYGANCiNJTklUSUFMSVpFDQpgYGB7cn0NCg0KDQojTnVtYmVyIG9mIHJlcGxpY2F0ZXMNCmsgPSAxMA0KZGF0YSA8LSByZGF0YQ0KI0VtcHR5IGxpc3QgYW5kIHZlY3RvcnMgZm9yIHJlcGxpY2F0ZXMNCmRDQ05saXN0IDwtIHJlcChsaXN0KG1hdHJpeChOQSwgbmNvbChyZGF0YSksIG5jb2wocmRhdGEpKSwgaykpDQpkUENDTmxpc3QgPC0gcmVwKGxpc3QobWF0cml4KE5BLCBuY29sKHJkYXRhKSwgbmNvbChyZGF0YSkpLCBrKSkNCkdHTWxpc3QgPC0gcmVwKGxpc3QobWF0cml4KE5BLCBuY29sKHJkYXRhKSwgbmNvbChyZGF0YSkpLCBrKSkNCnltYXQgPC0gbWF0cml4KDAsMTkxLGspDQpoZXIxIDwtIHJlcCgwLGspDQpyZGF0YXJlcHMgPC0gcmVwKGxpc3QobWF0cml4KE5BLCBuY29sKHJkYXRhKSwgbmNvbChyZGF0YSkpKSwgaykNCg0KI0VtcHR5IHZlY3RvcnMgZm9yIEFVQ1MgYW5kIHRoZWlyIGNvbmZpZGVuY2UgaW50ZXJ2YWxzIGNhbGN1bGF0ZWQgb3ZlciByZXBsaWNhdGVzDQoNCmRDQ05hdWMgPC0gcmVwKDAsaykNCmRQQ0NOYXVjPC0gcmVwKDAsaykNCnNpZ25kQ0NOYXVjPC0gcmVwKDAsaykNCnNpZ25kUENDTmF1YzwtIHJlcCgwLGspDQpleGhhdXN0aXZlYXVjIDwtIHJlcCgwLGspDQpkR0dNYXVjIDwtIHJlcCgwLGspDQoNCmRDQ05hdWMwMiA8LSByZXAoMCxrKQ0KZFBDQ05hdWMwMjwtIHJlcCgwLGspDQpzaWduZENDTmF1YzAyPC0gcmVwKDAsaykNCnNpZ25kUENDTmF1YzAyPC0gcmVwKDAsaykNCmV4aGF1c3RpdmVhdWMwMiA8LSByZXAoMCxrKQ0KZEdHTWF1YzAyIDwtIHJlcCgwLGspDQoNCiMjIyMjIyMjIyMjIyMjIyMjIyMjIyMjIyMjIyMjIyMjIyMjIyMjIyMjIyMjIyMjIw0KbG93LmNpLmRDQ05hdWMgPC0gcmVwKDAsaykNCmxvdy5jaS5kUENDTmF1YzwtIHJlcCgwLGspDQpsb3cuY2kuc2lnbmRDQ05hdWM8LSByZXAoMCxrKQ0KbG93LmNpLnNpZ25kUENDTmF1YzwtIHJlcCgwLGspDQpsb3cuY2kuZXhoYXVzdGl2ZWF1YyA8LSByZXAoMCxrKQ0KbG93LmNpLmRHR01hdWMgPC0gcmVwKDAsaykNCg0KbG93LmNpLmRDQ05hdWMwMiA8LSByZXAoMCxrKQ0KbG93LmNpLmRQQ0NOYXVjMDI8LSByZXAoMCxrKQ0KbG93LmNpLnNpZ25kQ0NOYXVjMDI8LSByZXAoMCxrKQ0KbG93LmNpLnNpZ25kUENDTmF1YzAyPC0gcmVwKDAsaykNCmxvdy5jaS5leGhhdXN0aXZlYXVjMDIgPC0gcmVwKDAsaykNCmxvdy5jaS5kR0dNYXVjMDIgPC0gcmVwKDAsaykNCiMjIyMjIyMjIyMjIyMjIyMjIyMjIyMjIyMjIyMjIyMjIyMjIyMjIyMjIyMjIyMjIw0KDQpoaWdoLmNpLmRDQ05hdWMgPC0gcmVwKDAsaykNCmhpZ2guY2kuZFBDQ05hdWM8LSByZXAoMCxrKQ0KaGlnaC5jaS5zaWduZENDTmF1YzwtIHJlcCgwLGspDQpoaWdoLmNpLnNpZ25kUENDTmF1YzwtIHJlcCgwLGspDQpoaWdoLmNpLmV4aGF1c3RpdmVhdWMgPC0gcmVwKDAsaykNCmhpZ2guY2kuZEdHTWF1YyA8LSByZXAoMCxrKQ0KDQpoaWdoLmNpLmRDQ05hdWMwMiA8LSByZXAoMCxrKQ0KaGlnaC5jaS5kUENDTmF1YzAyPC0gcmVwKDAsaykNCmhpZ2guY2kuc2lnbmRDQ05hdWMwMjwtIHJlcCgwLGspDQpoaWdoLmNpLnNpZ25kUENDTmF1YzAyPC0gcmVwKDAsaykNCmhpZ2guY2kuZXhoYXVzdGl2ZWF1YzAyIDwtIHJlcCgwLGspDQpoaWdoLmNpLmRHR01hdWMwMiA8LSByZXAoMCxrKQ0KDQojIyMjIyMjIyMjIyMjIyMjIyMjIyMjIyMjIyMjIyMjIyMjIyMjIyMjIyMjIyMjIyMjIyMjDQoNCg0KI0luZGljYXRlIHRoZSB0cnVlIHBvc2l0aXZlcw0KDQoNClJPQ21hdHJpeCA8LSBtYXRyaXgoMCxuY29sKHJkYXRhKSxuY29sKHJkYXRhKSkNCg0KUk9DbWF0cml4Wzc1LDE1MF0gPC0gMQ0KUk9DbWF0cml4WzEwMCwyMDBdIDwtIDENClJPQ21hdHJpeFsxMjUsMjE1XSA8LSAxDQpST0NtYXRyaXhbMjUsNTJdIDwtIDENCg0KUk9DbWF0cml4WzMzLDY2XSA8LSAxDQpST0NtYXRyaXhbODgsMTQ0XSA8LSAxDQoNClJPQ21hdHJpeFsyLDE3MF0gPC0gMQ0KUk9DbWF0cml4WzUwLDExNV0gPC0gMQ0KUk9DbWF0cml4WzQ0LDk5XSA8LSAxDQoNClJPQ21hdHJpeFsxMiwxODBdIDwtIDENClJPQ21hdHJpeFs2MCwxMjVdIDwtIDENClJPQ21hdHJpeFsyMiwxOTBdIDwtIDENClJPQ21hdHJpeFsyMiwyMTFdIDwtIDENCg0KDQoNClJPQ21hdHJpeFs3MCwxMzVdIDwtIDENClJPQ21hdHJpeFszMiwyMjFdIDwtIDENClJPQ21hdHJpeFsxMiwxODNdIDwtIDENClJPQ21hdHJpeFs1NCwxMDldIDwtIDENCg0KI1RydWUgcG9zaXRpdmUgZm9yIHRoZSBleGhhdXN0aXZlIHNlYXJjaA0KDQpvYnNlcnZhdGlvbnMgPC1yZWFkLmNzdigifi90cmFpbmluZ0RhdGEtcmVsZWFzZS5jc3YiLCBoZWFkZXIgPSBGQUxTRSkNCiAgb2JzZXJ2YXRpb25zIDwtIG9ic2VydmF0aW9uc1stMSxjKDQyOjI3MildDQogIG5hbXMgPC0gYXBwbHkoIGNvbWJuKGNvbG5hbWVzKG9ic2VydmF0aW9ucyksMiksIDIsIGZ1bmN0aW9uKHopIHBhc3RlKHosIGNvbGxhcHNlID0gJyonKSkNCiAgY29scyA8LSBjb21ibihuY29sKHJkYXRhKSwgMikNCiAgdGhyZWUgPC0gYXBwbHkoY29scywgMiwgZnVuY3Rpb24oeikgcm93UHJvZHMoYXMubWF0cml4KHJkYXRhKVssel0pKQ0KICBjb2xuYW1lcyh0aHJlZSkgPC0gbmFtcw0KICANCiAgDQpST0N2ZWMgPC0gcmVwKDAsZGltKHRocmVlKVsyXSkNCmN2Q29lZmZpY2llbnRzIDwtIG1hdHJpeCgwLGRpbSh0aHJlZSlbMl0saykNClJPQ3ZlY1t3aGljaChjb2xuYW1lcyh0aHJlZSkgPT0gIlYxMTYqVjE5MSIpXSA8LSAxDQpST0N2ZWNbd2hpY2goY29sbmFtZXModGhyZWUpID09ICJWMTQxKlYyNDEiKV0gPC0gMQ0KUk9DdmVjW3doaWNoKGNvbG5hbWVzKHRocmVlKSA9PSAiVjE2NipWMjU2IildIDwtIDENClJPQ3ZlY1t3aGljaChjb2xuYW1lcyh0aHJlZSkgPT0gIlY2NipWOTMiKV0gPC0gMQ0KDQoNClJPQ3ZlY1t3aGljaChjb2xuYW1lcyh0aHJlZSkgPT0gIlY3NCpWMTA3IildIDwtIDENClJPQ3ZlY1t3aGljaChjb2xuYW1lcyh0aHJlZSkgPT0gIlYxMjkqVjE4NSIpXSA8LSAxDQoNClJPQ3ZlY1t3aGljaChjb2xuYW1lcyh0aHJlZSkgPT0gIlY0MypWMjExIildIDwtIDENClJPQ3ZlY1t3aGljaChjb2xuYW1lcyh0aHJlZSkgPT0gIlY5MSpWMTU2IildIDwtIDENClJPQ3ZlY1t3aGljaChjb2xuYW1lcyh0aHJlZSkgPT0gIlY4NSpWMTQwIildIDwtIDENCg0KUk9DdmVjW3doaWNoKGNvbG5hbWVzKHRocmVlKSA9PSAiVjUzKlYyMjEiKV0gPC0gMQ0KUk9DdmVjW3doaWNoKGNvbG5hbWVzKHRocmVlKSA9PSAiVjEwMSpWMTY2IildIDwtIDENClJPQ3ZlY1t3aGljaChjb2xuYW1lcyh0aHJlZSkgPT0gIlY2MypWMjMxIildIDwtIDENClJPQ3ZlY1t3aGljaChjb2xuYW1lcyh0aHJlZSkgPT0gIlY2MypWMjUyIildIDwtIDENCg0KDQoNClJPQ3ZlY1t3aGljaChjb2xuYW1lcyh0aHJlZSkgPT0gIlYxMTEqVjE3NiIpXSA8LSAxDQpST0N2ZWNbd2hpY2goY29sbmFtZXModGhyZWUpID09ICJWNzMqVjI2MiIpXSA8LSAxDQpST0N2ZWNbd2hpY2goY29sbmFtZXModGhyZWUpID09ICJWNTMqVjIyNCIpXSA8LSAxDQpST0N2ZWNbd2hpY2goY29sbmFtZXModGhyZWUpID09ICJWOTUqVjE1MCIpXSA8LSAxDQoNCmBgYA0KI1NJTVVMQVRFIFRIRSBNT0RFTFMgQU5EIFBFUkZPUk0gQUxMIEFOQUxZU0VTDQpgYGB7cn0NCg0KIyMjIyMjIyMjIyMjIyMjIyMjIyMjU0lNVUxBVEVEIE1PREVMIyMjIyMjIyMjIyMjIyMjIyMjIyMjDQoNCg0KI1RXTyBSRUxVIFRFUk1TDQoNCnoxIDwtIHJkYXRhWywxMl0qcmRhdGFbLDE4M10NCnoyIDwtIHJkYXRhWywxMDldKnJkYXRhWyw1NF0NCg0KejFbd2hpY2goejEgPCBxdWFudGlsZSh6MSwgMC43NSkpXSA8LTANCnoyW3doaWNoKHoyIDwgcXVhbnRpbGUoejIsIDAuNzUpKV0gPC0wDQoNCg0KDQp5bWF0IDwtIG1hdHJpeCgwLDE5MSxrKQ0KZm9yKGwgaW4gMTprKXsNCiAgDQogIGRhdGEgPC1yZWFkLmNzdigicnIuY3N2IiwgaGVhZGVyID0gVFJVRSkNCiAgcmRhdGEgPC0gZGF0YVssYyg0MjoyNzIpXQ0KICANCiAgcmRhdGFbLDEyNV0gPC0gcmRhdGFbLDIxNV0gICsgcm5vcm0oMTkxLDAsMC4yNSkNCiAgcmRhdGFbLDc1XSA8LSByZGF0YVssMTUwXSArIHJub3JtKDE5MSwwLDAuMjUpDQogIA0KICANCiAgeW1hdFssbF0gPC0gcmRhdGFbLDc1XSpyZGF0YVssMTUwXStyZGF0YVssMTAwXSpyZGF0YVssMjAwXSArcmRhdGFbLDEyNV0qcmRhdGFbLDIxNV0gK3JkYXRhWywyNV0qcmRhdGFbLDUyXStyZGF0YVssMzNdKnJkYXRhWyw2Nl0rcmRhdGFbLDg4XSpyZGF0YVssMTQ0XSsNCiAgICAyKih6MSt6Mikrcm5vcm0oMTkxLDAsMS43NSkNCiAgeTEgPC0geW1hdFssbF0NCiAgaGVyMVtsXSA8LSAodmFyKHkxKS0xLjc1XjIpL3Zhcih5MSkNCiAgDQogIHJkYXRhW3doaWNoKHkxIDwgcXVhbnRpbGUoeTEsIDAuODApKSwyXSA8LSAtcmRhdGFbd2hpY2goeTEgPCBxdWFudGlsZSh5MSwgMC44MCkpLDE3MF0gKyBybm9ybShsZW5ndGgod2hpY2goeTEgPCBxdWFudGlsZSh5MSwgMC44MCkpKSwwLDAuMjUpDQogIHJkYXRhW3doaWNoKHkxIDwgcXVhbnRpbGUoeTEsIDAuODApKSw1MF0gPC0gLXJkYXRhW3doaWNoKHkxIDwgcXVhbnRpbGUoeTEsIDAuODApKSwxMTVdICsgcm5vcm0obGVuZ3RoKHdoaWNoKHkxIDwgcXVhbnRpbGUoeTEsIDAuODApKSksMCwwLjI1KQ0KICByZGF0YVt3aGljaCh5MSA+IHF1YW50aWxlKHkxLCAwLjgwKSksOTldIDwtIC1yZGF0YVt3aGljaCh5MSA+IHF1YW50aWxlKHkxLCAwLjgwKSksNDRdICsgcm5vcm0obGVuZ3RoKHdoaWNoKHkxID4gcXVhbnRpbGUoeTEsIDAuODApKSksMCwwLjI1KQ0KICANCiAgcmRhdGFbd2hpY2goeTEgPCBxdWFudGlsZSh5MSwgMC44MCkpLDEyXSA8LSByZGF0YVt3aGljaCh5MSA8IHF1YW50aWxlKHkxLCAwLjgwKSksMTgwXSArIHJub3JtKGxlbmd0aCh3aGljaCh5MSA8IHF1YW50aWxlKHkxLCAwLjgwKSkpLDAsMC4yNSkNCiAgcmRhdGFbd2hpY2goeTEgPCBxdWFudGlsZSh5MSwgMC44MCkpLDYwXSA8LSByZGF0YVt3aGljaCh5MSA8IHF1YW50aWxlKHkxLCAwLjgwKSksMTI1XSArIHJub3JtKGxlbmd0aCh3aGljaCh5MSA8IHF1YW50aWxlKHkxLCAwLjgwKSkpLDAsMC4yNSkNCiAgcmRhdGFbd2hpY2goeTEgPCBxdWFudGlsZSh5MSwgMC44MCkpLDIyXSA8LSByZGF0YVt3aGljaCh5MSA8IHF1YW50aWxlKHkxLCAwLjgwKSksMjExXSArIHJub3JtKGxlbmd0aCh3aGljaCh5MSA8IHF1YW50aWxlKHkxLCAwLjgwKSkpLDAsMC4yNSkNCiAgDQogIHJkYXRhW3doaWNoKHkxIDwgcXVhbnRpbGUoeTEsIDAuODApKSwyMl0gPC0gcmRhdGFbd2hpY2goeTEgPCBxdWFudGlsZSh5MSwgMC44MCkpLDE5MF0gKyBybm9ybShsZW5ndGgod2hpY2goeTEgPCBxdWFudGlsZSh5MSwgMC44MCkpKSwwLDAuMjUpDQogIHJkYXRhW3doaWNoKHkxIDwgcXVhbnRpbGUoeTEsIDAuODApKSw3MF0gPC0gcmRhdGFbd2hpY2goeTEgPCBxdWFudGlsZSh5MSwgMC44MCkpLDEzNV0gKyBybm9ybShsZW5ndGgod2hpY2goeTEgPCBxdWFudGlsZSh5MSwgMC44MCkpKSwwLDAuMjUpDQogIHJkYXRhW3doaWNoKHkxIDwgcXVhbnRpbGUoeTEsIDAuODApKSwzMl0gPC0gcmRhdGFbd2hpY2goeTEgPCBxdWFudGlsZSh5MSwgMC44MCkpLDIyMV0gKyBybm9ybShsZW5ndGgod2hpY2goeTEgPCBxdWFudGlsZSh5MSwgMC44MCkpKSwwLDAuMjUpDQogIA0KICB5bWF0WyxsXSA8LSB5bWF0WyxsXSArIDIqKHJkYXRhWywxMF0gKyByZGF0YVssMzBdICtyZGF0YVssNTBdICsgcmRhdGFbLDcwXSArIHJkYXRhWyw5MF0gKyByZGF0YVssMTAwXSkNCiAgeTEgPC0geW1hdFssbF0NCiAgDQogIA0KICBkYXRhIDwtIHJkYXRhDQogIHJkYXRhcmVwc1tbbF1dIDwtIHJkYXRhIA0KICANCiAgDQogIA0KICANCiAjIyMjI0VYSEFVU1RJVkUgU0VBUkNIIyMjIyMjIyMjIyMjIyMjIyMjIyMjIyMjDQogIA0KICAjRW51bWVyYXRlIGFsbCBwb3NzaWJsZSBwYWlyd2lzZSBpbnRlcmFjdGlvbnMNCiAgDQogIG9ic2VydmF0aW9ucyA8LSByZWFkLmNzdigifi90cmFpbmluZ0RhdGEtcmVsZWFzZS5jc3YiLCBoZWFkZXIgPSBUUlVFKQ0KICBvYnNlcnZhdGlvbnMgPC0gb2JzZXJ2YXRpb25zWy0xLGMoNDI6MjcyKV0NCiAgbmFtcyA8LSBhcHBseSggY29tYm4oY29sbmFtZXMob2JzZXJ2YXRpb25zKSwyKSwgMiwgZnVuY3Rpb24oeikgcGFzdGUoeiwgY29sbGFwc2UgPSAnKicpKQ0KICBjb2xzIDwtIGNvbWJuKG5jb2wocmRhdGEpLCAyKQ0KICB0aHJlZSA8LSBhcHBseShjb2xzLCAyLCBmdW5jdGlvbih6KSByb3dQcm9kcyhhcy5tYXRyaXgocmRhdGEpWyx6XSkpDQogIGNvbG5hbWVzKHRocmVlKSA8LSBuYW1zDQogIA0KICBjdi5maXQ9Y3YuZ2xtbmV0KHRocmVlLCB5MSwgYWxwaGEgPSAwLCBtYXhpdCA9IDEwMDAwKQ0KICBmaXQ9Z2xtbmV0KHRocmVlLHkxLCBhbHBoYSA9IDAsIG1heGl0ID0gMTAwMDApDQogIGN2Q29lZmZpY2llbnRzWyxsXSA8LSBjb2VmKGZpdCwgcyA9IGN2LmZpdCRsYW1iZGEubWluKVstMV0NCiAgDQogIA0KICANCiAgDQogIA0KICANCiAgDQogICMjIyMjIyMjIyMjI2RpZmZlcmVudGlhbCBHR00jIyMjIyMjIyMNCiAgaGlnaCA8LSByZGF0YVt3aGljaCh5MSA+IHF1YW50aWxlKHkxLCAwLjUpKSxdDQogIGxvdyA8LSByZGF0YVt3aGljaCh5MSA8IHF1YW50aWxlKHkxLCAwLjUpKSxdDQogIGZnbC5yZXN1bHRzID0gSkdMKFk9bGlzdChsb3csIGhpZ2gpLHBlbmFsdHk9ImZ1c2VkIixsYW1iZGExPS4xLGxhbWJkYTI9LjEscmV0dXJuLndob2xlLnRoZXRhPVRSVUUpDQogIEdHTSA8LWFicyhhcy5tYXRyaXgoZmdsLnJlc3VsdHMkdGhldGFbWzFdXSktYXMubWF0cml4KGZnbC5yZXN1bHRzJHRoZXRhW1syXV0pKQ0KICANCiAgDQogICMjIyMjIyMjIyMjIyMjIyMjIyMjIyMjIw0KICAjIyNkaWZmZXJlbnRpYWwgRENDTiMjIyMNCiAgZENDTiA8LSBhYnMoY29yKGhpZ2gpIC0gY29yKGxvdykpDQogIHNpZ25kQ0NOIDwtIChhYnMoc2lnbihjb3IoaGlnaCkpLXNpZ24oY29yKGxvdykpKSkqZENDTg0KICANCiAgIyMjIyMjIyMjIyMjIyMjIyMjIyMjIyMjDQogIA0KICANCiAgDQogIA0KICANCiAgDQogICMjIyMjIyMjIyMjI1NpZ24tYWRqdXN0ZWQgZFBDQ04jIyMjIyMjIyMjIyMjIyMjIw0KICANCiAgI1Jlc2lkdWFsIHN0ZXANCiAgcmRhdGEgPC0gYXMubWF0cml4KHJkYXRhKQ0KICBjdi5maXQgPC0gY3YuZ2xtbmV0KHJkYXRhLCBzY2FsZSh5MSksIGFscGhhID0gMS4wLCBtYXhpdCA9IDEwMDAwKQ0KICBmaXQ9Z2xtbmV0KHJkYXRhLCBzY2FsZSh5MSksIGFscGhhID0gMS4wLCBtYXhpdCA9IDEwMDAwKQ0KICBDb2VmZmljaWVudHMgPC0gY29lZihmaXQsIHMgPSBjdi5maXQkbGFtYmRhLm1pbikNCiAgdiA8LSByZGF0YSAlKiUgQ29lZmZpY2llbnRzWy0xXQ0KICByZXNpZCA8LSBzY2FsZSh5MSktdg0KICANCiAgDQogICNFc3RpbWF0ZSBkUENDTiBzdHJ1Y3R1cmVzDQogIGEgPSAwLjENCiAgZFBDQ04gPC0gbWF0cml4KDAsbmNvbChkYXRhKSxuY29sKGRhdGEpKQ0KICBzaWduUCA8LSBtYXRyaXgoMCxuY29sKGRhdGEpLG5jb2woZGF0YSkpDQogIA0KICANCiAgZm9yKGkgaW4gMTpuY29sKGRhdGEpKXsNCiAgICBmb3IoaiBpbiAxOm5jb2woZGF0YSkpew0KICAgICAgDQogICAgICByZXMgPC0gKGxtKHJkYXRhWyxqXX5yZGF0YVssaV0pJHJlc2lkdWFscykNCiAgICAgIHNldCA8LSBjYmluZChyZXNpZCxyZXMscmRhdGFbLGldKQ0KICAgICAgaGlnaCA8LSBzZXRbd2hpY2gocmVzaWQgPiBxdWFudGlsZShyZXNpZCwgMS8yKSksXQ0KICAgICAgbG93IDwtIHNldFt3aGljaChyZXNpZCA8IHF1YW50aWxlKHJlc2lkLDEvMikpLF0NCiAgICAgIGhpZ2ggPC0gaGlnaFssLTFdDQogICAgICBsb3cgPC0gbG93WywtMV0NCiAgICAgIA0KICAgICAgDQogICAgICBpZihhYnMoY29yKGhpZ2hbLDFdLGhpZ2hbLDJdKSkgPCBhKXsNCiAgICAgICAgaCA8LSAwDQogICAgICB9ZWxzZXsNCiAgICAgICAgaCA8LSBjb3IoaGlnaFssMV0saGlnaFssMl0pDQogICAgICB9DQogICAgICANCiAgICAgIGlmKGFicyhjb3IobG93WywxXSxsb3dbLDJdKSkgPCBhKXsNCiAgICAgICAgTCA8LSAwDQogICAgICB9ZWxzZXsNCiAgICAgICAgTCA8LSBjb3IobG93WywxXSxsb3dbLDJdKQ0KICAgICAgfQ0KICAgICAgDQogICAgICBkUENDTltpLGpdIDwtIGgtTA0KICAgICAgDQogICAgICBzaWduUFtpLGpdIDwtIDAuNSphYnMoKHNpZ24oaCktc2lnbihMKSkpDQogICAgICANCiAgICAgIA0KICAgIH0NCiAgfQ0KICANCiAgZGlhZyhkUENDTikgPC0gMA0KICANCiAgc2lnblBbc2lnblAgIT0gMCBdIDwtIDENCiAgc2lnbmRQQ0NOIDwtIGFicyhzaWduUCpkUENDTikNCiAgDQogIA0KDQogIA0KICAjQ2FsY3VsYXRlIEFVQ1MgZm9yIGVhY2ggcmVwbGljYXRpb24NCiAgDQogIGRDQ05hdWMwMltsXSA8LSByb2MoUk9DbWF0cml4W3VwcGVyLnRyaShST0NtYXRyaXgpXSxkQ0NOW3VwcGVyLnRyaShkQ0NOKV0sIHBhcnRpYWwuYXVjID0gYygwLjgsMS4wKSwgcGFydGlhbC5hdWMuY29ycmVjdCA9IFRSVUUpJGF1Yw0KICBkUENDTmF1YzAyW2xdPC0gcm9jKFJPQ21hdHJpeFt1cHBlci50cmkoUk9DbWF0cml4KV0sZFBDQ05bdXBwZXIudHJpKGRQQ0NOKV0sIHBhcnRpYWwuYXVjID0gYygwLjgsMS4wKSwgcGFydGlhbC5hdWMuY29ycmVjdCA9IFRSVUUpJGF1Yw0KICBzaWduZENDTmF1YzAyW2xdPC0gcm9jKFJPQ21hdHJpeFt1cHBlci50cmkoUk9DbWF0cml4KV0sc2lnbmRDQ05bdXBwZXIudHJpKHNpZ25kQ0NOKV0sIHBhcnRpYWwuYXVjID0gYygwLjgsMS4wKSwgcGFydGlhbC5hdWMuY29ycmVjdCA9IFRSVUUpJGF1Yw0KICBzaWduZFBDQ05hdWMwMltsXTwtIHJvYyhST0NtYXRyaXhbdXBwZXIudHJpKFJPQ21hdHJpeCldLHNpZ25kUENDTlt1cHBlci50cmkoc2lnbmRQQ0NOKV0sIHBhcnRpYWwuYXVjID0gYygwLjgsMS4wKSwgcGFydGlhbC5hdWMuY29ycmVjdCA9IFRSVUUpJGF1Yw0KICBleGhhdXN0aXZlYXVjMDJbbF0gPC0gcm9jKFJPQ3ZlYyxhYnMoY3ZDb2VmZmljaWVudHNbLGxdKSxwYXJ0aWFsLmF1YyA9IGMoMC44LDEuMCksIHBhcnRpYWwuYXVjLmNvcnJlY3QgPSBUUlVFKSRhdWMNCiAgDQogIGRDQ05hdWNbbF0gPC0gcm9jKFJPQ21hdHJpeFt1cHBlci50cmkoUk9DbWF0cml4KV0sZENDTlt1cHBlci50cmkoZENDTildKSRhdWMNCiAgZFBDQ05hdWNbbF08LSByb2MoUk9DbWF0cml4W3VwcGVyLnRyaShST0NtYXRyaXgpXSxkUENDTlt1cHBlci50cmkoZFBDQ04pXSkkYXVjDQogIHNpZ25kQ0NOYXVjW2xdPC0gcm9jKFJPQ21hdHJpeFt1cHBlci50cmkoUk9DbWF0cml4KV0sc2lnbmRDQ05bdXBwZXIudHJpKHNpZ25kQ0NOKV0pJGF1Yw0KICBzaWduZFBDQ05hdWNbbF08LSByb2MoUk9DbWF0cml4W3VwcGVyLnRyaShST0NtYXRyaXgpXSxzaWduZFBDQ05bdXBwZXIudHJpKHNpZ25kUENDTildKSRhdWMNCiAgZXhoYXVzdGl2ZWF1Y1tsXSA8LSByb2MoUk9DdmVjLCBhYnMoY3ZDb2VmZmljaWVudHNbLGxdKSkkYXVjDQogIA0KICANCiAgZEdHTWF1Y1tsXSA8LSAgcm9jKFJPQ21hdHJpeFt1cHBlci50cmkoUk9DbWF0cml4KV0sR0dNW3VwcGVyLnRyaShhcy5tYXRyaXgoR0dNKSldKSRhdWMNCiAgZEdHTWF1YzAyW2xdIDwtICByb2MoUk9DbWF0cml4W3VwcGVyLnRyaShST0NtYXRyaXgpXSxHR01bdXBwZXIudHJpKGFzLm1hdHJpeChHR00pKV0sIHBhcnRpYWwuYXVjID0gYygwLjgsMS4wKSwgcGFydGlhbC5hdWMuY29ycmVjdCA9IFRSVUUpJGF1Yw0KICANCiAgDQogIA0KIyMjIyMjIyMjIyMjIyMjIyMjIyMjIyMjIyMjIyMjIyMjIyMjIyMjIw0KICANCiAgDQogIGxvdy5jaS5kQ0NOYXVjMDJbbF0gPC0gY2kuYXVjKHJvYyhST0NtYXRyaXhbdXBwZXIudHJpKFJPQ21hdHJpeCldLGRDQ05bdXBwZXIudHJpKGRDQ04pXSwgcGFydGlhbC5hdWMgPSBjKDAuOCwxLjApLCBwYXJ0aWFsLmF1Yy5jb3JyZWN0ID0gVFJVRSkkYXVjKVsxXQ0KICBsb3cuY2kuZFBDQ05hdWMwMltsXTwtIGNpLmF1Yyhyb2MoUk9DbWF0cml4W3VwcGVyLnRyaShST0NtYXRyaXgpXSxkUENDTlt1cHBlci50cmkoZFBDQ04pXSwgcGFydGlhbC5hdWMgPSBjKDAuOCwxLjApLCBwYXJ0aWFsLmF1Yy5jb3JyZWN0ID0gVFJVRSkkYXVjKVsxXQ0KICBsb3cuY2kuc2lnbmRDQ05hdWMwMltsXTwtIGNpLmF1Yyhyb2MoUk9DbWF0cml4W3VwcGVyLnRyaShST0NtYXRyaXgpXSxzaWduZENDTlt1cHBlci50cmkoc2lnbmRDQ04pXSwgcGFydGlhbC5hdWMgPSBjKDAuOCwxLjApLCBwYXJ0aWFsLmF1Yy5jb3JyZWN0ID0gVFJVRSkkYXVjKVsxXQ0KICBsb3cuY2kuc2lnbmRQQ0NOYXVjMDJbbF08LSBjaS5hdWMocm9jKFJPQ21hdHJpeFt1cHBlci50cmkoUk9DbWF0cml4KV0sc2lnbmRQQ0NOW3VwcGVyLnRyaShzaWduZFBDQ04pXSwgcGFydGlhbC5hdWMgPSBjKDAuOCwxLjApLCBwYXJ0aWFsLmF1Yy5jb3JyZWN0ID0gVFJVRSkkYXVjKVsxXQ0KICBsb3cuY2kuZXhoYXVzdGl2ZWF1YzAyW2xdIDwtIGNpLmF1Yyhyb2MoUk9DdmVjLGFicyhjdkNvZWZmaWNpZW50c1ssbF0pLHBhcnRpYWwuYXVjID0gYygwLjgsMS4wKSwgcGFydGlhbC5hdWMuY29ycmVjdCA9IFRSVUUpJGF1YylbMV0NCiAgDQogIGxvdy5jaS5kQ0NOYXVjW2xdIDwtIGNpLmF1Yyhyb2MoUk9DbWF0cml4W3VwcGVyLnRyaShST0NtYXRyaXgpXSxkQ0NOW3VwcGVyLnRyaShkQ0NOKV0pJGF1YylbMV0NCiAgbG93LmNpLmRQQ0NOYXVjW2xdPC0gY2kuYXVjKHJvYyhST0NtYXRyaXhbdXBwZXIudHJpKFJPQ21hdHJpeCldLGRQQ0NOW3VwcGVyLnRyaShkUENDTildKSRhdWMpWzFdDQogIGxvdy5jaS5zaWduZENDTmF1Y1tsXTwtIGNpLmF1Yyhyb2MoUk9DbWF0cml4W3VwcGVyLnRyaShST0NtYXRyaXgpXSxzaWduZENDTlt1cHBlci50cmkoc2lnbmRDQ04pXSkkYXVjKVsxXQ0KICBsb3cuY2kuc2lnbmRQQ0NOYXVjW2xdPC0gY2kuYXVjKHJvYyhST0NtYXRyaXhbdXBwZXIudHJpKFJPQ21hdHJpeCldLHNpZ25kUENDTlt1cHBlci50cmkoc2lnbmRQQ0NOKV0pJGF1YylbMV0NCiAgbG93LmNpLmV4aGF1c3RpdmVhdWNbbF0gPC0gY2kuYXVjKHJvYyhST0N2ZWMsIGFicyhjdkNvZWZmaWNpZW50c1ssbF0pKSRhdWMpWzFdDQogIA0KICANCiAgbG93LmNpLmRHR01hdWNbbF0gPC0gIGNpLmF1Yyhyb2MoUk9DbWF0cml4W3VwcGVyLnRyaShST0NtYXRyaXgpXSxHR01bdXBwZXIudHJpKGFzLm1hdHJpeChHR00pKV0pJGF1YylbMV0NCiAgbG93LmNpLmRHR01hdWMwMltsXSA8LSAgY2kuYXVjKHJvYyhST0NtYXRyaXhbdXBwZXIudHJpKFJPQ21hdHJpeCldLEdHTVt1cHBlci50cmkoYXMubWF0cml4KEdHTSkpXSwgcGFydGlhbC5hdWMgPSBjKDAuOCwxLjApLCBwYXJ0aWFsLmF1Yy5jb3JyZWN0ID0gVFJVRSkkYXVjKVsxXQ0KICANCiAgDQojIyMjIyMjIyMjIyMjIyMjIyMjIyMjIyMjIyMjIyMjIyMjIyMjIyMjIyMjIyMjIyMjIyMjIyMjDQogIA0KICBoaWdoLmNpLmRDQ05hdWMwMltsXSA8LSBjaS5hdWMocm9jKFJPQ21hdHJpeFt1cHBlci50cmkoUk9DbWF0cml4KV0sZENDTlt1cHBlci50cmkoZENDTildLCBwYXJ0aWFsLmF1YyA9IGMoMC44LDEuMCksIHBhcnRpYWwuYXVjLmNvcnJlY3QgPSBUUlVFKSRhdWMpWzNdDQogIGhpZ2guY2kuZFBDQ05hdWMwMltsXTwtIGNpLmF1Yyhyb2MoUk9DbWF0cml4W3VwcGVyLnRyaShST0NtYXRyaXgpXSxkUENDTlt1cHBlci50cmkoZFBDQ04pXSwgcGFydGlhbC5hdWMgPSBjKDAuOCwxLjApLCBwYXJ0aWFsLmF1Yy5jb3JyZWN0ID0gVFJVRSkkYXVjKVszXQ0KICBoaWdoLmNpLnNpZ25kQ0NOYXVjMDJbbF08LSBjaS5hdWMocm9jKFJPQ21hdHJpeFt1cHBlci50cmkoUk9DbWF0cml4KV0sc2lnbmRDQ05bdXBwZXIudHJpKHNpZ25kQ0NOKV0sIHBhcnRpYWwuYXVjID0gYygwLjgsMS4wKSwgcGFydGlhbC5hdWMuY29ycmVjdCA9IFRSVUUpJGF1YylbM10NCiAgaGlnaC5jaS5zaWduZFBDQ05hdWMwMltsXTwtIGNpLmF1Yyhyb2MoUk9DbWF0cml4W3VwcGVyLnRyaShST0NtYXRyaXgpXSxzaWduZFBDQ05bdXBwZXIudHJpKHNpZ25kUENDTildLCBwYXJ0aWFsLmF1YyA9IGMoMC44LDEuMCksIHBhcnRpYWwuYXVjLmNvcnJlY3QgPSBUUlVFKSRhdWMpWzNdDQogIGhpZ2guY2kuZXhoYXVzdGl2ZWF1YzAyW2xdIDwtIGNpLmF1Yyhyb2MoUk9DdmVjLGFicyhjdkNvZWZmaWNpZW50c1ssbF0pLHBhcnRpYWwuYXVjID0gYygwLjgsMS4wKSwgcGFydGlhbC5hdWMuY29ycmVjdCA9IFRSVUUpJGF1YylbM10NCiAgDQogIGhpZ2guY2kuZENDTmF1Y1tsXSA8LSBjaS5hdWMocm9jKFJPQ21hdHJpeFt1cHBlci50cmkoUk9DbWF0cml4KV0sZENDTlt1cHBlci50cmkoZENDTildKSRhdWMpWzNdDQogIGhpZ2guY2kuZFBDQ05hdWNbbF08LSBjaS5hdWMocm9jKFJPQ21hdHJpeFt1cHBlci50cmkoUk9DbWF0cml4KV0sZFBDQ05bdXBwZXIudHJpKGRQQ0NOKV0pJGF1YylbM10NCiAgaGlnaC5jaS5zaWduZENDTmF1Y1tsXTwtIGNpLmF1Yyhyb2MoUk9DbWF0cml4W3VwcGVyLnRyaShST0NtYXRyaXgpXSxzaWduZENDTlt1cHBlci50cmkoc2lnbmRDQ04pXSkkYXVjKVszXQ0KICBoaWdoLmNpLnNpZ25kUENDTmF1Y1tsXTwtIGNpLmF1Yyhyb2MoUk9DbWF0cml4W3VwcGVyLnRyaShST0NtYXRyaXgpXSxzaWduZFBDQ05bdXBwZXIudHJpKHNpZ25kUENDTildKSRhdWMpWzNdDQogIGhpZ2guY2kuZXhoYXVzdGl2ZWF1Y1tsXSA8LSBjaS5hdWMocm9jKFJPQ3ZlYywgYWJzKGN2Q29lZmZpY2llbnRzWyxsXSkpJGF1YylbM10NCiAgDQogIA0KICBoaWdoLmNpLmRHR01hdWNbbF0gPC0gIGNpLmF1Yyhyb2MoUk9DbWF0cml4W3VwcGVyLnRyaShST0NtYXRyaXgpXSxHR01bdXBwZXIudHJpKGFzLm1hdHJpeChHR00pKV0pJGF1YylbM10NCiAgaGlnaC5jaS5kR0dNYXVjMDJbbF0gPC0gIGNpLmF1Yyhyb2MoUk9DbWF0cml4W3VwcGVyLnRyaShST0NtYXRyaXgpXSxHR01bdXBwZXIudHJpKGFzLm1hdHJpeChHR00pKV0sIHBhcnRpYWwuYXVjID0gYygwLjgsMS4wKSwgcGFydGlhbC5hdWMuY29ycmVjdCA9IFRSVUUpJGF1YylbM10NCiAgDQogIA0KfQ0KDQoNCmBgYA0KI0FWRVJBR0UgVEhFIFJFU1VMVFMgT1ZFUiBSRVBMSUNBVElPTlMNCmBgYHtyfQ0KYyhtZWFuKGRDQ05hdWMwMiksIG1lYW4obG93LmNpLmRDQ05hdWMwMiksbWVhbihoaWdoLmNpLmRDQ05hdWMwMikpDQpjKG1lYW4oc2lnbmRDQ05hdWMwMixuYS5ybT1UUlVFKSwgbWVhbihsb3cuY2kuc2lnbmRDQ05hdWMwMiksbWVhbihoaWdoLmNpLnNpZ25kQ0NOYXVjMDIpKQ0KYyhtZWFuKHNpZ25kUENDTmF1YzAyKSwgbWVhbihsb3cuY2kuc2lnbmRQQ0NOYXVjMDIpLG1lYW4oaGlnaC5jaS5zaWduZFBDQ05hdWMwMikpDQpjKG1lYW4oZXhoYXVzdGl2ZWF1YzAyLG5hLnJtPVRSVUUpLCBtZWFuKGxvdy5jaS5leGhhdXN0aXZlYXVjMDIpLG1lYW4oaGlnaC5jaS5leGhhdXN0aXZlYXVjMDIpKQ0KYyhtZWFuKGRHR01hdWMwMiksIG1lYW4obG93LmNpLmRHR01hdWMwMiksbWVhbihoaWdoLmNpLmRHR01hdWMwMikpDQoNCmMobWVhbihkQ0NOYXVjKSwgbWVhbihsb3cuY2kuZENDTmF1YyksbWVhbihoaWdoLmNpLmRDQ05hdWMpKQ0KYyhtZWFuKHNpZ25kQ0NOYXVjKSwgbWVhbihsb3cuY2kuc2lnbmRDQ05hdWMpLG1lYW4oaGlnaC5jaS5zaWduZENDTmF1YykpDQpjKG1lYW4oc2lnbmRQQ0NOYXVjKSwgbWVhbihsb3cuY2kuc2lnbmRQQ0NOYXVjKSxtZWFuKGhpZ2guY2kuc2lnbmRQQ0NOYXVjKSkNCmMobWVhbihleGhhdXN0aXZlYXVjKSwgbWVhbihsb3cuY2kuZXhoYXVzdGl2ZWF1YyksbWVhbihoaWdoLmNpLmV4aGF1c3RpdmVhdWMpKQ0KYyhtZWFuKGRHR01hdWMpLCBtZWFuKGxvdy5jaS5kR0dNYXVjKSxtZWFuKGhpZ2guY2kuZEdHTWF1YykpDQpgYGANCg0KLS0tDQoNCg==
