# Supplementary material for: Model guided trait-specific co-expression network estimation as a new perspective for identifying molecular interactions and pathways
Source: PLoS Comput Biol. 2021 May 3;17(5):e1008960. doi: 10.1371/journal.pcbi.1008960 (PMC8118548; doi:10.1371/journal.pcbi.1008960)
Supplement: S2 Appendix — See also a GitHub repository https://github.com/JAJKontio/model_diffnet.git. (ZIP) [file pcbi.1008960.s002.zip › Preview_documents/Toyexample_preview.pdf]

Rdocumentation: model_diffnet

#Install required R-packages.

```
install.packages("glmnet",repos = "http://cran.us.r-project.org");library("glmnet"
install.packages("matrixStats",repos = "http://cran.us.r-project.org");library("ma
install.packages("qgraph",repos = "http://cran.us.r-project.org");library("qgraph"
```

#Example of the sign-adjusted dCCN method.

#Simulate a simple model consisting of two main effects, two type I interactions, and one type II interaction.

```
#At first, simulate 1000 i.i.d samples of ten explanatory variables.
X <- matrix(0,1000,10)
for(i in 1:10){
  X[,i] <- rnorm(1000,0,1)}

#Let us consider a scenario, where the resistance for a particular disease is cont
resistance <- X[,1]*X[,5] + X[,2]*X[,8] + rnorm(1000,0,0.5)

#Suppose that there exist a regulatory relationship between genes X[,9] and X[,10]

X[which(resistance > quantile(resistance, 0.5)),10] <- X[which(resistance > quanti

#Let us also assume that, among individuals with low resistance-levels, this regul

X[which(resistance < quantile(resistance, 0.5)),10] <-  rnorm(500,0,1)
X[which(resistance < quantile(resistance, 0.5)),9] <-  rnorm(500,0,1)


# Finally, simulate the trait associated with that particular disease as the sum c
y <- X[,3] + X[,7] + resistance

#Now the proposed model guided differential network estimation method can be used
```

#Apply the proposed model_diffnet function

```
source("./model_diffnet.R")
dCCN_diff_net <- model_diffnet(X,y,cut_off_a = 1/2,alpha = 1/3,r = 0.1, corr_metri
qgraph(dCCN_diff_net)
```

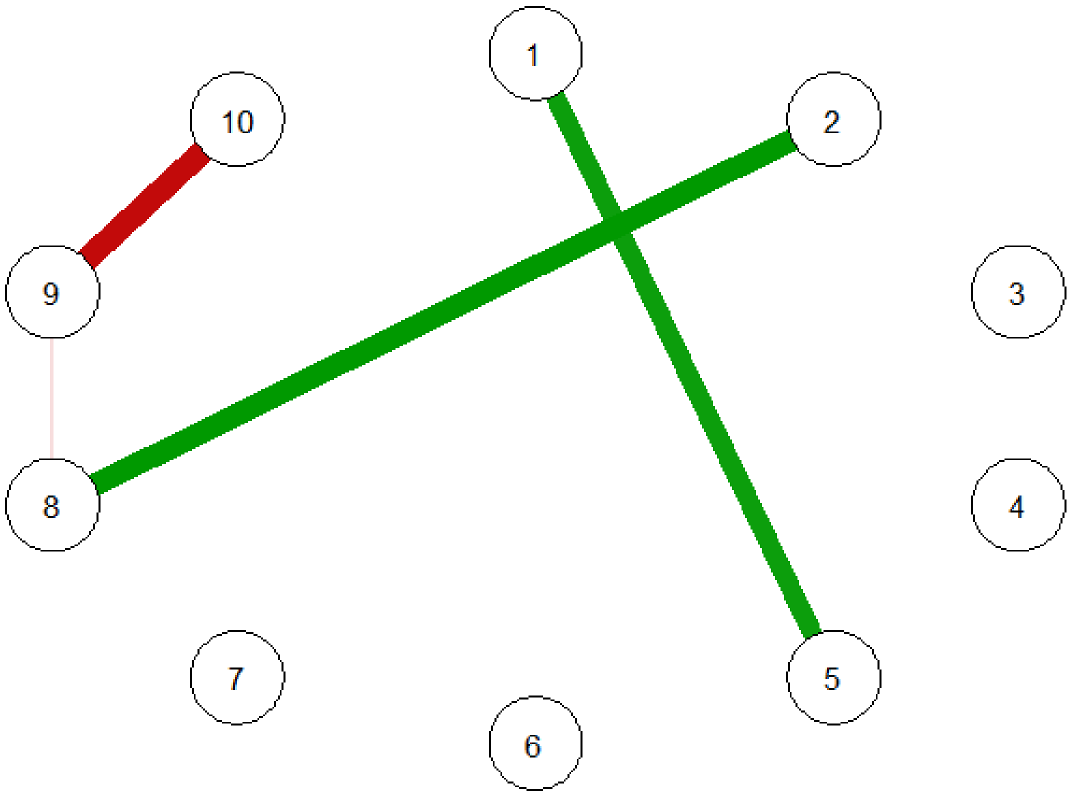

dCCN_diff_net

```
##              [,1]      [,2] [,3] [,4]      [,5] [,6] [,7]       [,8]       [,9]
##  [1,] 0.0000000 0.0000000    0    0 0.9253421    0    0  0.0000000  0.0000000
##  [2,] 0.0000000 0.0000000    0    0 0.0000000    0    0  0.9702755  0.0000000
##  [3,] 0.0000000 0.0000000    0    0 0.0000000    0    0  0.0000000  0.0000000
##  [4,] 0.0000000 0.0000000    0    0 0.0000000    0    0  0.0000000  0.0000000
##  [5,] 0.9253421 0.0000000    0    0 0.0000000    0    0  0.0000000  0.0000000
##  [6,] 0.0000000 0.0000000    0    0 0.0000000    0    0  0.0000000  0.0000000
##  [7,] 0.0000000 0.0000000    0    0 0.0000000    0    0  0.0000000  0.0000000
##  [8,] 0.0000000 0.9702755    0    0 0.0000000    0    0  0.0000000 -0.1283770
##  [9,] 0.0000000 0.0000000    0    0 0.0000000    0    0 -0.1283770  0.0000000
## [10,] 0.0000000 0.0000000    0    0 0.0000000    0    0  0.0000000 -0.9309715
##             [,10]
##  [1,]  0.0000000
##  [2,]  0.0000000
##  [3,]  0.0000000
##  [4,]  0.0000000
##  [5,]  0.0000000
##  [6,]  0.0000000
##  [7,]  0.0000000
##  [8,]  0.0000000
##  [9,] -0.9309715
## [10,]  0.0000000
```

#Example of the sign-adjusted dPCCN method. (a more complex type II interaction scenario) #Simulate a simple model consisting of two main effects, two type I interactions, and one type II interaction.

```
#Simulate 1000 i.i.d samples of ten explanatory variables.
X <- matrix(0,1000,10)
for(i in 1:10){
  X[,i] <- rnorm(1000,0,1)}

#Let us consider the same scenario as above, and simulate the resistance vector as
resistance <- X[,1]*X[,5] + X[,2]*X[,8] + rnorm(1000,0,0.5)

#Now let us consider a more complex type II interaction scenario, where genes X[,9
X[which(resistance < quantile(resistance, 0.5)),10] <-  rnorm(500,0,0.1)
X[which(resistance < quantile(resistance, 0.5)),9] <-  rnorm(500,0,0.1)

#Now suppose  that this "stable", low-variance behaviour of gene X[,9] is assumed
X[which(resistance > quantile(resistance, 0.5)),9] <- rnorm(500,0,1)

#Let us also assume that, among individuals with low resistance-levels, gene X[,9]
X[which(resistance > quantile(resistance, 0.5)),10] <- X[which(resistance > quanti


#Simulate the final trait associated with that particular disease as the sum of th
y <- X[,3] + X[,7] + resistance
```

#Apply the proposed model_diffnet function

```
source("./model_diffnet.R")
diff_net <- model_diffnet(X,y,cut_off_a = 1/2,alpha = 1/3,r = 0.1, corr_metric = "
qgraph(diff_net)
```

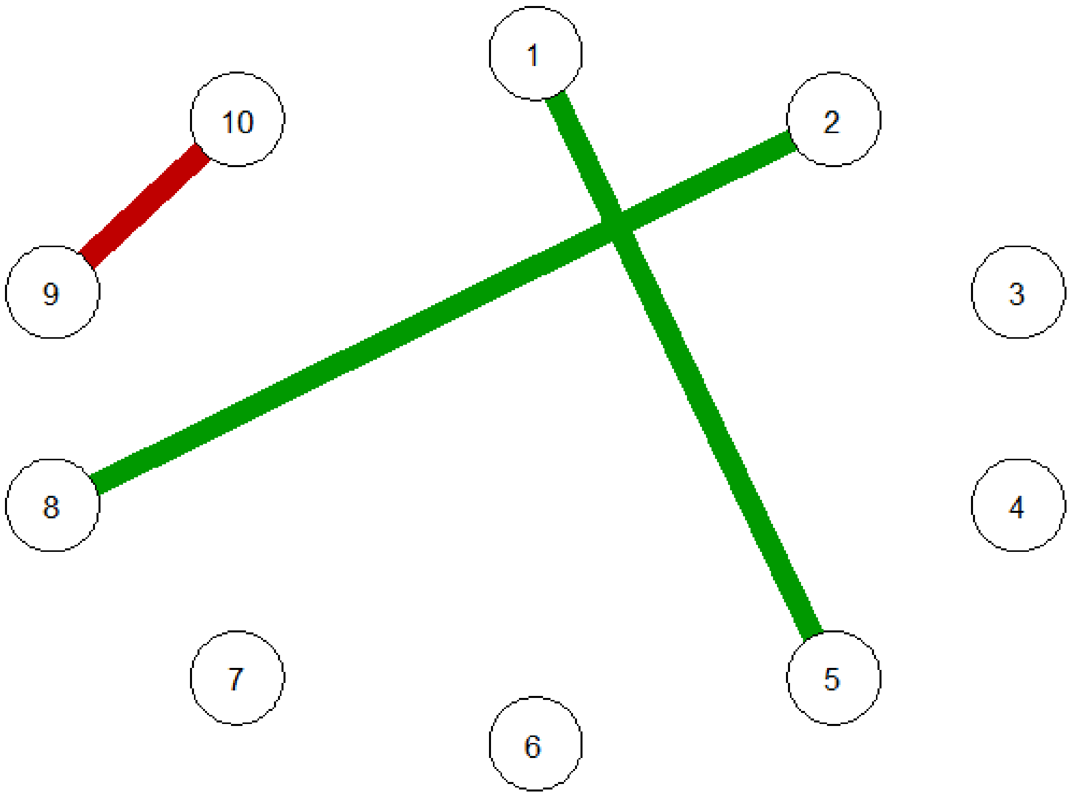


```
diff_net
```

```
##       [,1] [,2] [,3] [,4] [,5] [,6] [,7] [,8] [,9] [,10]
##  [1,]    0    0    0    0    1    0    0    0    0     0
##  [2,]    0    0    0    0    0    0    0    1    0     0
##  [3,]    0    0    0    0    0    0    0    0    0     0
##  [4,]    0    0    0    0    0    0    0    0    0     0
##  [5,]    1    0    0    0    0    0    0    0    0     0
##  [6,]    0    0    0    0    0    0    0    0    0     0
##  [7,]    0    0    0    0    0    0    0    0    0     0
##  [8,]    0    1    0    0    0    0    0    0    0     0
##  [9,]    0    0    0    0    0    0    0    0    0    -1
## [10,]    0    0    0    0    0    0    0    0   -1     0
```
