# Supplementary material for: Model guided trait-specific co-expression network estimation as a new perspective for identifying molecular interactions and pathways
Source: PLoS Comput Biol. 2021 May 3;17(5):e1008960. doi: 10.1371/journal.pcbi.1008960 (PMC8118548; doi:10.1371/journal.pcbi.1008960)
Supplement: S1 Table — Estimation is done over patients with non-censored survival times (142 individuals) in the DREAM9 AML dataset. (PDF) [file pcbi.1008960.s003.pdf]

The Aiken-West interaction test results regarding interactions CCNE1:AKT1 and ASNS:GAB2 in the acute myeloid leukemia example analysis are shown in S1 Table.

S1 Table. Estimated effect sizes, standard errors and p-values for the main and interaction effects on survival times in the Aiken-West interaction test. Estimation is done over patients with non-censored survival times (142 individuals) in the DREAM9 AML dataset.

Proteins	Estimate	Std. Error	t value	Pr(>|t|)	Proteins	Estimate	Std. Error	t value	Pr(>|t|)
CCNE1	-0.2113	0.0784	-2.70	0.0079	ASNS	-0.1237	0.1213	-1.02	0.3095
AKT1	0.1297	0.0946	1.37	0.1725	GAB2	-0.1050	0.0832	-1.26	0.2091
CCNE1:AKT1	-0.4742	0.1081	-4.39	$1.0 \cdot 10^{-5}$	ASNS:GAB2	0.3444	0.0932	3.69	0.0003
