# Supplementary material for: Model guided trait-specific co-expression network estimation as a new perspective for identifying molecular interactions and pathways
Source: PLoS Comput Biol. 2021 May 3;17(5):e1008960. doi: 10.1371/journal.pcbi.1008960 (PMC8118548; doi:10.1371/journal.pcbi.1008960)
Supplement: Attachment — Submitted filename: Author_response_to_review_comments.pdf [file pcbi.1008960.s004.pdf]

Authors' Response to Reviews of

Model guided trait-specific co-expression network estimation as a new perspective for identifying molecular interactions and pathways

Juho A. J. Kontio, Tanja Pyhäjärvi and Mikko J. Sillanpää
PLoS Computational Biology, PCOMPBIOL-D-20-01509

RC: ***Reviewers' Comment***, AR: Authors' Response

Summary

We would like to thank all reviewers for their insightful comments and efforts to improve our work – they have tremendously increased the quality of this manuscript. The common concern of all reviewers was that the spelling of the manuscript was too technical spelling to be appealing for a broad readership. All reviewers have suggested to explain the paper's content in a more intuitive light with a schematic diagram of the approach. The revised version includes two figures that 1) represent the overall idea (Fig. 1) and the major steps of the provided method (Fig. 2).

After reading the reviewers comments, we wholeheartedly agree that this was the most fundamental problem of the previous version. After completion the corresponding edits, the general quality of the manuscript has tremendously increased and much wider audience can be now reached. All the changes in the revised manuscript are highlighted with yellow color and the removed text is simply overlined.

Prof. Mikko J. Sillanpää on behalf of all authors

1. Reviewer #1

RC: ***The manuscript is in general clearly written but not accessible to non-specialists due to many technical details of the method that lack intuitive explanation. I did not find obvious flaws in the methodology and derivations, and justification has been provided for individual steps in the algorithm. The overall description would benefit from a more intuitive explanation for instance in the form of an illustration. Some of the benchmarking details could be moved to supplementary material in order to improve readability.***

AR: The underlying thought here was well-founded, and with that in mind, we entirely agreed after revising the manuscript. It truly suffered from too technical spelling to be appealing for a broad readership. Therefore, inspired by your suggestions, we thoroughly re-wrote the first section to summarize the paper's content in a more intuitive light (lines 49 - 75). In particular, the revised version includes schematic representations for the overall idea (Fig. 1) and the logical structure underlying the provided algorithm (Fig. 2). These diagrams has the same three-fold layout as the text structure (lines 49 - 65 and lines 160 - 199), and thus, quickly guide readers through the technicalities if necessary.

We would like to thank you for bringing up these valuable suggestions. They tremendously improved the comprehensibility and general quality of the manuscript.

RC: ***The most important explanations for the improved performance of the proposed method could be more***

clearly stated in Discussion.

AR: As the matter of fact, following your first suggestion, we believe that these re-written parts already provide a concrete explanation for the considerable advance of our approach over these reference methods (Fig 1).

1.1. Major concern

RC: ***Observed co-expression provides evicence of association rather than interaction. Please reconsider the terminology or justify the use of the term "interactions".***

AR: Thank you for noting that this terminology might be confusing at first glance. An explanation for this diction is now stated more clearly in the manuscript (lines 142 - 146) to avoid such semantic confusion: It is evident that co-expression networks, *per se*, could provide only evidence of associations between genes. Thus, referring such network structures as "interactions" would be nothing but the misuse of terminology. However, rather than viewing co-expression networks as standalone concepts, we reminded readers of their role in the provided methodological fusion (see also the new figure 1). The co-expression networks are – within this framework – always bound to be trait-specific *via* the underlying parametric interaction model. What they provide, as a consequence, is exactly the evidence of interactions (type I and II) in light of this underlying model.

*Note that the same justification should clarify the meaning if you were referring the issue of *guilt-by-association* and related conlusive misinterpretations.

RC: ***The proposed method appears to perform well but the overall novelty is limited since a number of co-expression techniques have been introduced over the last 20 years.***

AR: It is true that countless co-expression network techniques have been introduced over the decades, and various parametric interaction search algorithms even longer. In both cases, the vast amount of research devoted to the algorithmic challenges has yielded the state-of-art methodologies that are nearly impossible to contribute with substantial improvements. On the other hand, this is actually the very premise motivating our work, *as the synergistic potential of these methodologies has remained completely unnoticed* (Figure 1). This is now stated clearly already in the abstract and further emphasized throughout the first section.

However, we have to respectively disagree with your statement about the novelty of this work, and would like to explain our differing opinion in turn. *This method is not merely just another co-expression technique, nor a standalone parametric interaction search algorithm. It is a fused combination of them that cross-exploits the strengths of both approaches in the estimation process.* The majority of papers under this topic only focus on improving the power and/or scalability of methods scheme-specifically. In this work, all deficiencies we are considering are more fundamental and mostly unresolved as they are totally detached from the above-mentioned computational properties. We both underscored and methodologically formalized a conceptual shift required for moving beyond these fundamental restrictions. In other words, instead of racing with these state-of-art methods scheme-specifically, we provided a totally new perspective by which we reached that substantial improvement in performance after all. This can be seen in the simulated examples.

RC: ***Although source codes are shared in the supplementary material, this work is not delivering a general-purpose ready-to-use implementation. This forms a major limitation for practical application and further benchmarking in other studies.***

Thank you for bringing up your concern about this – we have now improved this work considerably on that matter. A toy-example is now available in the supplementary materials. All R-codes will be available in the GitHub repository immediately after a possible acceptance (as they are now in the supplementary

materials). They will include an R-function that executes the whole procedure in an autotamic manner as a ready-to-use implementation. To enable an straightforward user-specific customization of this, we also decided to implement step-specific functions as well. Moreover, these individual functions are shown in the workflow diagram (Fig. 2) to associate them with the procedural flow (displayed in it) for elucidation.

Minor

l. 41 "A little" -> "Little". l. 277: paper -> work? l. 447: ".," -> ","

Table 1: the best performing methods (numeric values) could be highlighted (bolded) in order to facilitate interpretation

AR: All of these minor issues are changed accordingly in the revised manuscript. We would like to use this opportunity to thank you for your reviewing time, effort and expertise by which you contributed to improve the manuscript to a great extent.

2. Reviewer #2

The authors present a method for interaction network inference, which combines features of co-expression networks and parametric interaction models. The idea is quite beautiful at its simplicity, which, however, is somewhat hard to grasp while reading the first parts of the manuscript. Therefore, some improvement in the flow of Abstract and Introduction Sections is warranted. Code availability should be guaranteed.

Major Comments:

RC: ***Please explain more concretely already in the abstract, what the paper's achievement is. Now, I felt that I had to read up to page 6-7 to get an understanding this. The description "framework for parallel consideration of parametric interaction models with quantitative traits and co-expression networks based on a previously uncharacterized link between them" should be made more tangible, in my opinion.***

AR: The previous version of the manuscript truly stagnated in motivating the problem. We truly appreciate your feedback on this – the changes against these suggestions made the manuscript of much better quality and more effortless to read. Now the manuscript quickly cuts to the chase by pointing out the overall conceptual idea more concretely (lines 49 - 75).

RC: ***A schematic diagram of the approach could make the paper accessible to a wider audience. This could include a general illustration of: how differential co-expression networks work and how parametric interaction models work, what is the link between them and how your work utilizes this. The Objectives on page 6 are an excellent summary of the goal. If these came into the illustration as well, we would have an overview of the idea in the paper shown also to a wider audience.***

AR: This was a tremendously valuable suggestion, as the spelling was indeed too technical to be appealing for a wider audience (see also the first comment/answer of Reviewer #1). Therefore, by following your idea, we prepared two schematic diagrams illustrating 1) the paper's conceptual design and premises, as well as 2) the methodological structure underlying the procedure itself. To serve as a reference guide through the technicalities, these diagrams are layouted consistently in line with the same text structure where the concepts are formally presented (lines 49 - 75 and lines 147 - 198).

RC: ***Please go through the manuscript and reduce complexity in paragraphs, where there is a subordinate***

clause in the middle of a paragraph without it having been made distinct with punctuation. For example, "For instance, in transcriptional interactions where a transcription factor binds to promoter regions of a particular gene to regulate its expression levels can be disrupted in cancers" is hard to grasp without reading several times. At simplest, add helpful punctuation, as in "For instance, in transcriptional interactions*,* where a transcription factor binds to promoter regions of a particular gene to regulate its expression*,* levels can be disrupted in cancers". Refactoring, though, would in most cases lead to a better result than this simplest suggestion. Especially the Introduction Section suffers from a high load of this type of complex paragraphs.

AR: We have now spell-checked the manuscript and simplified the overall complexity of sentences and paragraphs on a need basis.

RC: ***I did not find the S2 Appendix or "Data and software" Supplement, where the code was referred to be found. Code availability is key for a method's impact. It would be highly beneficial, if the code was available in an accessible form, for instance, as an R/Bioconductor package, or at least on GitHub or similar repository. This would give the authors also a platform to making further work with the tool.***

AR: A toy-example is now available in the supplementary materials. All R-codes will be available in the GitHub repository immediately after a possible acceptance (as they are now in the supplementary materials). They will include an R-function that executes the whole procedure in an autotamic manner as a ready-to-use implementation. To enable an straightforward user-specific customization of this, we also decided to implement step-specific functions as well. Moreover, these individual functions are shown in the workflow diagram (Fig. 2) to associate them with the procedural flow (displayed in it) for elucidation.

RC: ***I am not convinced that it is appropriate to estimate the confidence intervals of a method with the ci.auc function of the pROC package. Should you not be bootstrapping the original data before model fitting instead of bootstrapping the predictions after model fitting? Won't the latter approach lead to a great overconfidence as the bootstrapping takes place only after model fitting?***

AR: Thank you for this technical note, and now that you mention it, there seems to be a possibility for getting overconfident intervals indeed. Therefore, reported intervals (or more precisely the way they are produced) might open more questions than clarifying anything. They are now removed from the table for simplicity, and AUCs are reported conventionally to avoid possible confusion.

RC: ***I do not understand, why the authors are omitting the right-censored observations from the study. As the authors describe, it is not conventional and it leads to a biased inference of the hazard function. Wouldn't it have been just simpler and more appropriate for the paper to include all observations? Could you provide a sensitivity analysis regarding the omission of observations?***

AR: This is a valid point and should be addressed carefully – this is just a matter of technical simplicity and will lead to bias estimates no matter the arguments. We have now stated more clearly (lines 313 - 328) in the manuscript that revealing the most important features of this data is not the primary task here, and we are fully aware that some aspects might seem unreasonable from that perspective. This example serves to provide one kind of practical example of cross-exploiting the strengths of individual schemes to overcome the deficiencies of one and another. Therefore, we chose to use intentionally a particularly problematic dataset, which also might seem controversial on occasion.

RC: ***Why is elastic net estimator used in the real data experiment, whereas L1/LASSO is used in the simulated data experiments? The choice should be motivated. Furthermore, it would be beneficial to provide a way forward regarding which method should the reader choose, if one were to use you're approach for new data.***

AR: Thanks for catching and pointing out this little blemish. We have now re-evaluated the method with the elastic net using the weight $\alpha = 1/3$ for consistency, and as expected, the results did not change much. To be honest, the use of this arbitrarily chosen value is nothing but a personal convention. There is no reason to prefer this over LASSO ($\alpha = 1$) or the Ridge penalty ($\alpha = 0$). The issue of searching for some sort of "optimal" α-value is a bit irrelevant on this matter. As such, this 1/3 is now a pre-fixed default value in the provided R-code, but can be easily changed user-specifically.

RC: ***Combined risk classifier: Why are only low- and high-risk patients included in the combined risk classifier, whereas all were included in the single-interaction classifiers? For consistency, result should be presented for complete data, too.***

AR: In our humble opinion, we believe that only these two risk-classes have the parametric interpretations that are worth for considering in this paper. The reason is the same that revealing the most important features of this data is not the primary task here, and this is the very issue, that might seem unreasonable from that perspective. The problem is that the proportion of survival time variation that can be captured by one classifier, in relation to other classifier, is unknown and its evaluation is beyond the scope of this paper. As such, we aim to improve the readability of the results section by simplifying things at this point.

RC: ***Minor Comments:***

- **Abstract: Clarify paragraph: what is referred to by “them”?**
- **Figure 1: Colors in the figure are not friendly for color blind.**
- **p.18,l.431: Wording with “consistently” is perhaps overstatement for a p-value of 0.083.**

AR: All of these minor issues are changed accordingly in the revised manuscript, and more color-blind friendly image will be uploaded after acceptance. We would like to thank you for your insightful comments and efforts to improve our work. They have tremendously increased the quality of this manuscript.

3. Reviewer #3

Thanks for an interesting idea and a nice work! The authors present a new method to identify important gene-gene interactions with respect to the relationship between expression and certain phenotypes. Specifically, their model combines parametric interactions with those represented by co-expression networks. They show that this type of model uncovers biologically meaningful interactions and allows for better associations with phenotypes such as disease survival time.

The proposed method builds conceptually on existing methods in interesting ways, and seems to be a promising tool for biological discovery. My comments are mainly in regards to strengthening and clarifying the framing and validation of the method.

Comments:

RC: ***It seems that DREAM9 data analysis involved protein expression levels, while the TCGA/GTEx data analysis involved RNA expression levels. Since RNA and protein levels are affected differently by regulation, often do not correlate highly, and provide different information about biological state, this distinction should be discussed. Is this method better suited toward one or the other? Since interactions derived from***

protein levels were validated using RNA-seq expression levels, does that suggest that the same important interactions can be discovered using either one?

AR: This is a valid point and we have now emphasized the differences between the DREAM9 protein-expression dataset and TCGA gene-expression dataset used only for validating the results (lines 382 - 388). However, we think that finding the interactions in both RNA and protein dataset is conservative, implying that this difference will not change our main conclusions.

RC: ***This varies from person to person and is not essential, but I would benefit from a figure containing a diagrammatic overview of the method. It could show what kind of data it uses, how it uses it to model interactions, what information it produces, and how that information could be applied to e.g. prognosis.***

AR: This has been a tremendously valuable suggestion received from all reviewers, and we are entirely in agreement. The manuscript now includes schematic representations of the paper's conceptual idea (Fig. 1) and the overview of the algorithmic implementation itself (Fig. 2). Furthermore, these diagrams are designed to quickly guide readers through the technicalities if needed.

RC: ***Validation of type II interactions was done using data from TCGA and GTEx projects via GEPIA. My understanding is that GTEx individuals did not have AML or any disease survival times. The authors should clarify how survival data was used in relation to the AML and non-AML individuals in this combined group.***

AR: Thanks for pointing this out as the GEPIA-software (http://gepia.cancer-pku.cn/about.html) was relatively new to us. Our misconception was due to fact that this software is built upon both TCGA and GTEx databases, and it can be quickly seen that this survival plot tool do have AML individuals with measured survival times as well. However, it seems that this web-tool automatically uses TCGA-database for survival analysis and GTEx individuals for other. We would like to thank you for informing about this, and it has been now clarified in the manuscript.

RC: ***Are there prognostic results from the DREAM9 challenge that this method can be compared to? Or is the main point more about the type of interactions uncovered (which presumably were not uncovered by the DREAM9 challenge participants) more so than the magnitude of prognostic ability?***

AR: The results might be available upon request, but as you said, our primary focus is to demonstrate the relationships between these two methodological schemes. Thank you for your suggestion, this comparison would be interesting indeed. However, as it is beyond the scope of this work, we believe that this additional comparison might cause unnecessary confusion.

RC: ***I was not provided with the R code mentioned in the paper, but I trust that it will be provided with the publication to help people run the method.***

AR: A toy-example is now available in the supplementary materials. All R-codes will be available in the GitHub repository immediately after a possible acceptance (as they are now in the supplementary materials). They will include an R-function that executes the whole procedure in an autotamic manner as a ready-to-use implementation. To enable an straightforward user-specific customization of this, we also decided to implement step-specific functions as well. Moreover, these individual functions are shown in the workflow diagram (Fig. 2) to associate them with the procedural flow (displayed in it) for elucidation.
