# Supplementary material for: Model guided trait-specific co-expression network estimation as a new perspective for identifying molecular interactions and pathways
Source: PLoS Comput Biol. 2021 May 3;17(5):e1008960. doi: 10.1371/journal.pcbi.1008960 (PMC8118548; doi:10.1371/journal.pcbi.1008960)
Supplement: Attachment — Submitted filename: Author_response_to_review_comments_05042021 (1).pdf [file pcbi.1008960.s005.pdf]

Authors' Response to Reviews of

Model guided trait-specific co-expression network estimation as a new perspective for identifying molecular interactions and pathways

Juho A. J. Kontio, Tanja Pyhäjärvi and Mikko J. Sillanpää
PLoS Computational Biology, PCOMPBIOL-D-20-01509R1

RC: ***Reviewers' Comment***, AR: Authors' Response

Summary

We would like to thank all reviewers for their valuable comments and huge efforts to improve our work and the quality of this manuscript. The majority of our work on this revision was devoted for improving the software and its usability which is now available in the GitHub repository https://github.com/JAJKontio/model_diffnet and released with the **GNU General Public License v3.0**. This is mentioned in lines 191 and 728. Only minor changes are made to the manuscript: We noticed a notational overlap and corrected it *(lines 605-607, 638 and 640)*. We also clarified the difference between two alternative metrics that can be used in the proposed method *(lines 599, 642-643 and 685-689)*. Also, Figure 2 is now changed to be coherent with the published R-function.

1. Reviewer #1

RC: ***Thank you for the improvements. The motivation and technique is now more clearly explained, and reproducible source code provided.***

Minor remaining comments:

1: I would like to verify that the reproducible source code contains also all code to replicate figures, tables and other such results represented in the manuscript.

2: Is an open source license (such as MIT) associated with the code - which license?

If reproducible code for the main results is provided (and not only a demo example of the method itself), and the code is released with an open license for fluent verification and reuse, then I have no further suggestions.

AR: Software and simulated replicates are now available in the supplementary materials as well as in the GitHub repository https://github.com/JAJKontio/model_diffnet released with the **GNU General Public License v3.0**. It contains the complete code for the provided analysis of acute myeloid leukemia cohort (which is available upon registration at https://www.synapse.org/#!Synapse:syn2455683/wiki/64007), simulated examples, and even more improved tutorial for the method itself using a within-code simulated toy example dataset. Moreover, an R-function *model_diffnet* is now available in the GitHub repository which runs the whole procedure in an autotamic manner as a ready-to-use implementation for users that are less experienced with R-language.

2. Reviewer #3

The authors have sufficiently addressed my comments.
